# Liver quad culture chip as a model for radiation injury research

**DOI:** 10.1038/s41598-025-96140-1

**Published:** 2025-04-11

**Authors:** Yuki Ueda, Satoshi Omiya, Jonathan Pinney, Michelle A. Bylicky, Molykutty J. Aryankalayil

**Affiliations:** 1 Radiation Oncology Branch, Center for Cancer Research, National Cancer Institute, 10 Center Drive, Room B3B406, Bethesda, MD 20892 USA; 2 Gryphon Scientific, Takoma Park, MD 20912 USA

**Keywords:** Biological models, Cell biology

## Abstract

Both cancer patients receiving radiotherapy and civilians in a mass casualty nuclear event may suffer from radiation induced damage to organ systems. Radiation induced liver disease (RILD) can cause acute and long-term organ dysfunction that potentially leads to death. The objective of this study was to ascertain the validity of a liver quad-culture chip, a micro-physiological system comprising primary human hepatocytes and non-parenchymal cells (NPCs), including liver sinusoidal endothelial cells, hepatic stellate cells (HSCs), and Kupffer cells, as a model for RILD. The radiation exposure to the chip model resulted in DNA damage and cellular senescence of hepatocytes and NPCs. We observed metabolic dysfunction, inflammation, endothelial dysfunction, and HSCs activation. Whole genome sequencing revealed gene alterations in pathways relevant to RILD, as well as the potential efficacy of *N*-acetylcysteine amide (NACA) against RILD. NACA exhibited the capacity to mitigate DNA damage and cellular senescence and decreased the impact of radiation exposure on other pathophysiological changes. CDKN1A and miR-34a-5p were validated as useful radiation response and treatment efficacy biomarkers. These findings highlight the potential of the liver quad-culture chip as an effective model for investigating the microenvironment in RILD and for evaluating the efficacy of therapeutic countermeasures and biomarkers.

## Introduction

**Fig. 1:**
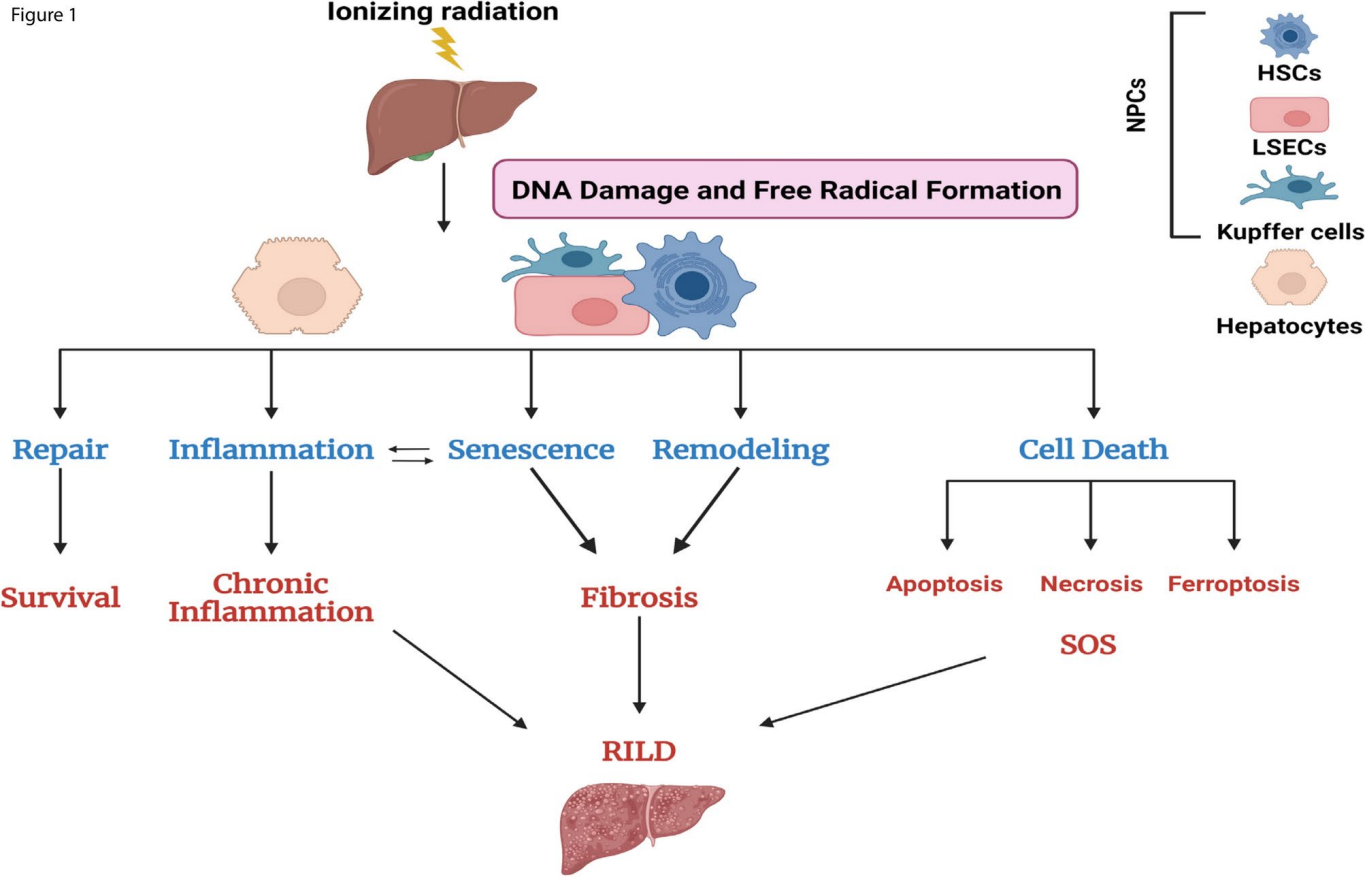
Schematic illustration of liver response to radiation injury and altered gene expression in our liver quad-culture chip model. Radiation injury induces direct DNA damage and indirect damage through the production of reactive oxygen species which may further damage DNA and other macromolecules including lipids and proteins in the cell. Hepatocytes and NPCs must repair DNA breaks to survive. But this survival may be marked by increases in inflammation which forms a positive feedback loop with senescence. Tissue remodeling may occur as dead and damaged cells are replaced, in the short term these changes may be beneficial, but can also lead to fibrosis. Cell death may occur for cells that fail to repair, and this cell death may include ferroptosis, necrosis, and apoptosis, among other pathways.

Radiation-induced liver disease (RILD) represents a significant concern for cancer patients undergoing radiation therapy and for civilians exposed to radiation in the event of a nuclear or radiological incident. RILD is a limiting factor in the application of radiation therapy not only for the treatment of liver cancer but also for total body irradiation in preparation for allogeneic bone marrow or hematopoietic stem cell transplantation; the liver is the most affected organ after total body irradiation^1–5^. Radiation exposure can cause acute and chronic dysfunction of the liver including RILD which is marked by hepatomegaly, the development of ascites, fibrosis, cirrhosis and, in some cases, death one to three months after radiation therapy. Development of RILD is dependent on several factors including: liver volume irradiated, dose, co-morbid conditions, and primary liver cancer, with 6–66% of patients showing symptoms after fractionated doses between 30 and 60 Gy^6–9^. Patients with chronic liver disease, such as viral hepatitis or cirrhosis, exhibit more acute and severe dysregulation of liver function and a more severe clinical course^9–11^. Radiation injury to the liver is thought to induce dysfunction through multiple mechanisms. One of the pathologies of RILD is characterized by sinusoidal obstruction syndrome (SOS) in which liver sinusoids in central areas become blocked; this condition is life threatening. The initial event in the development of SOS is considered to be the death and dysfunction of liver sinusoidal endothelial cells (LSECs), which constitute the blood vessels in the liver. LSECs injury results in the onset of inflammation, impairment of the endothelial barrier, fibrin deposition and thrombus formation^12–14^. Consequently, the disruption of microcirculatory blood flow and the hypoxic environment ultimately leads to the death of hepatocytes. Furthermore, hepatic steatosis and fibrosis are frequently observed in cases of hepatic irradiation. Hepatocytes undergo cellular senescence and send out inflammatory signals to the surrounding tissue. Hepatic stellate cells (HSCs) become activated and induce collagen accumulation and tissue remodeling. Kupffer cells play a pivotal role in inflammation and the innate immune response. Kupffer cells may die and contribute to LSECs injury^12–14^. Radiation induced pathophysiology is shown (Fig. 1).

Currently treatments for RILD are symptom-focused, as there is no established diagnostic method, and no specific therapeutic drug is available to reverse or repair injury^15^. Defibrotide is currently the only potential treatment for patients who have developed SOS. It has mostly been used to treat patients who received radiation for hematopoietic stem cell transplant; there is no current, agreed upon treatment protocol^16^. Further, even with treatment, patient survival at day 100 after SOS development was 58%^17^, indicating a need for improved treatment. Therefore, there is a tremendous need for the development of diagnostic and therapeutic countermeasures against RILD.

Previous studies of radiation induced liver injury have shown the potential importance of mRNA, microRNA (miRNA) and long non-coding RNA (lncRNA) in pathophysiology and post-exposure dose determination using animal models^5,18,19^. However, each animal model from mouse to primate features limitations which must be considered, including: differential dose sensitivities, failure to reproduce clinical pathophysiology of RILD, and subsequent failure to translate data produced in these animal studies into relevant treatment options for patients^20–22^. Two-dimensional monolayer cultures of primary endothelial cells have traditionally been used to complement problems of animal models. However, these static cell culture systems are unable to simulate the complex conditions observed in vivo, including cell–cell interactions, cell–matrix interactions, and shear flow stress conditions. Organoid cultures taken from cancer patients and used to predict treatment efficacy may be a useful approach for personalized medicine; but their high failure rate (10/33 samples) in establishing initial organoids suggests improvements are needed^23^. Additionally, there are limitations in the ability of human pluripotent stem cells (hPSCs) to develop into the needed cell types present in adults^24^. While Kupffer cells and hepatic stellate cells have been successfully generated from hPSCs, LSECs could not be produced in this manner^25^. Since LSECs play a crucial role in the development of SOS and RILD, this limitation hampers the ability of organoid cultures to accurately model the liver’s response to radiation.

Recently, micro-physiological systems (MPS), also called organ-on-a-chip technology, have been developed as an in vitro system that better mimics the in vivo microenvironment^26,27^. The liver quad-culture chip demonstrated better predictive sensitivity for drug-induced liver injury compared to 3D hepatic spheroids^26^ . We have previously published the impact of radiation injury on a liver co-culture chip model comprising solely of hepatocytes and LSECs^28^. We reported distinct differences in expression patterns between hepatocytes and LSECs and the utility of analyzing these cells separately. Nevertheless, we did not observe significant LSECs injury or markers of fibrosis.This indicated that the absence of these responses may be attributed to a deficiency in Kupffer cells and HSCs, other common cell types of the liver. Inhibition of Kupffer cells with gadolinium chloride prior to radiation led to lower IL-1b and TNF-α mRNA expression in liver samples at 6 h, as well as decreased LSEC apoptosis 24 h post 30 Gy radiation in a murine model^29^. HSCs isolated from mice exhibited upregulation of *Ccl5* and *p21* mRNA and secreted CCL5 protein at day 7 post 3.8 Gy radiation injury in vitro^30^. These findings suggest that both cell types play a role in LSEC death and the subsequent development of RILD after radiation, prompting further investigation into their roles in our MPS model.

The purpose of this study is to highlight the utility of the liver quad-culture chip (hepatocytes, LSECs, Kupffer cells, HSCs) as an effective model for studying RILD. Here, we demonstrated that this liver quad-culture chip model can be used to analyze gene expression alterations and RNA biomarkers relevant to the pathophysiology of RILD in hepatocytes and non-parenchymal cells (NPCs: LSECs, Kupffer cells, HSCs), including DNA damage, cellular senescence, cell death, inflammation and fibrosis. We identified *N*-acetylcysteine amide (NACA) as an effective therapeutic countermeasure for RILD through pathway-specific analysis of our whole genome analysis using Ingenuity Pathway Analysis (IPA) and demonstrated its efficacy in alleviating key pathophysiological changes in NPCs and hepatocytes that lead to RILD.

## Results

### The liver quad-culture chip characterization and response to radiation injury

**Fig. 2:**
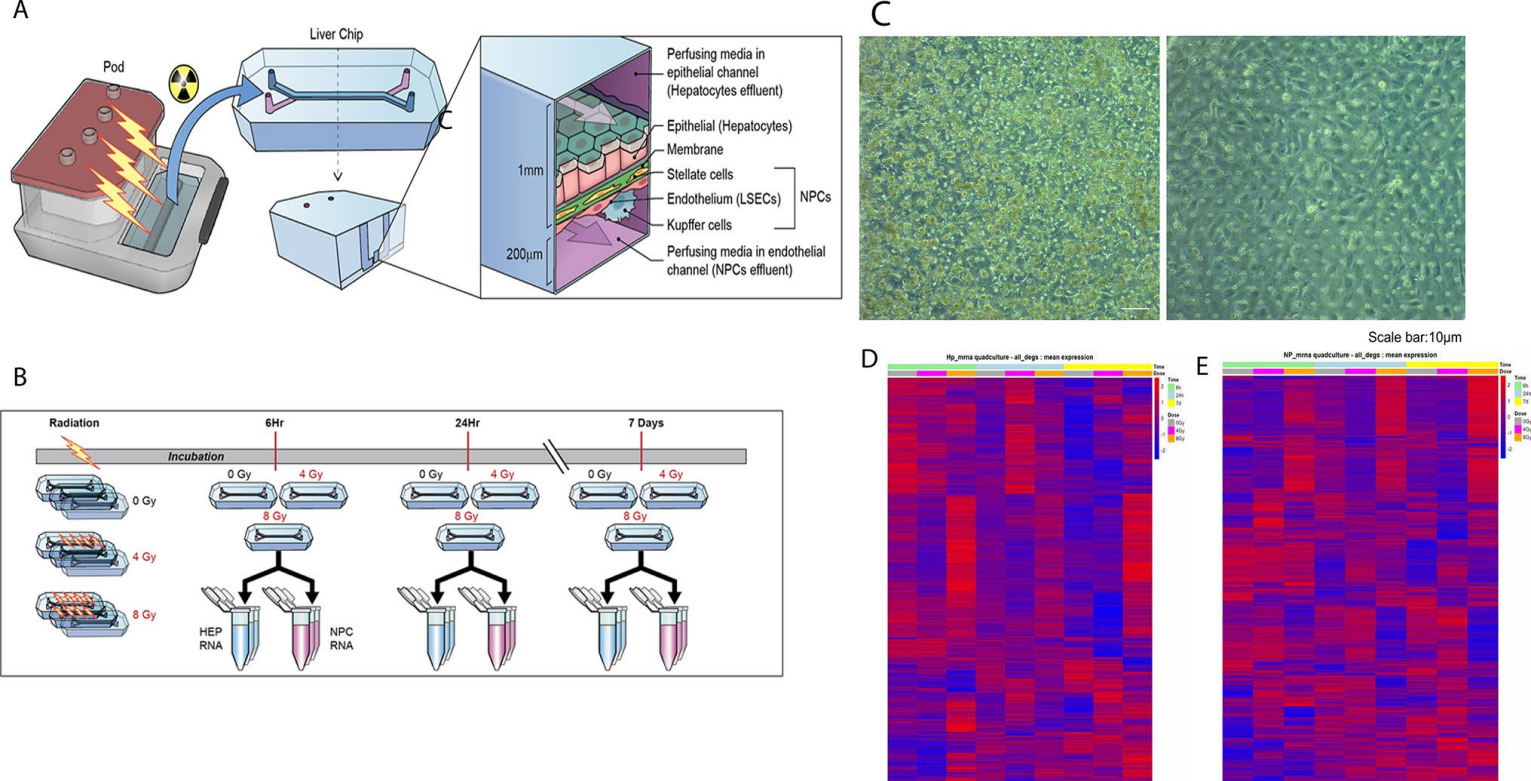
Characterization of the liver quad-culture chip after radiation injury. (**A**) The Emulate Liver quad-culture chip in which hepatocytes are grown on the upper channel while LSECs, HSCs and Kupffer cells are grown on the lower channel. These channels are separated by a semi-permeable membrane which allows communication among the groups but does not allow cell migration from one channel to the other. Each channel receives separate perfusing media at a defined and controllable flow rate which replicates shear stress in normal liver. (**B**) RNA collection after radiation injury at 6 h, 24 h and 7d post 0 Gy, 4 Gy or 8 Gy radiation. (**C**) Hepatocytes and NPCs in quad-culture chip using bright-field microscopy. (**D**) and (**E**) Hepatocytes and NPCs heatmaps across all doses and times where red indicates upregulation and blue indicates downregulation compared to the mean. The highest bars represent time: Green represents 6 h, light blue represents 24 h and yellow represent 7d. The second bars represent dose where gray represents 0 Gy, pink indicates 4 Gy and orange indicates 8 Gy.

The liver-on-a-chip model from Emulate consists of two chambers which contain the four main cell types of the liver. In our model hepatocytes were attached to the upper epithelial chamber while NPCs were attached to the lower endothelial chamber (Fig. 2A,B). Continuous flow of media occurs through both chambers to mimic the stress that cells anticipate in the body. Radiation doses were chosen based on dose response curves. The expression of key genes in our liver co-culture manuscript^28^ were validated in our liver quad-culture chip at 4 Gy, 6 Gy, 8 Gy and 10 Gy; the greatest alteration in gene expression was observed at 8 Gy (Supplemental Figure S1). Consequently, we focused on 4 Gy and 8 Gy for this study. Cells received a single dose of 4 Gy, 8 Gy or sham irradiation (0 Gy) and were collected for RNA extraction at 6 h, 24 h and 7 days post irradiation and sent for whole genome RNA sequencing (Fig. 2C). This is further discussed in the Methods section. Briefly, hepatocytes and NPCs grew on separate chambers and were lysed directly in their channels then isolated separately. We performed whole genome sequencing on NPCs and hepatocytes. We could not separate NPCs into their respective cell types and so analyzed them as a group. Volcano plots for each dose, time and cell type for mRNA and lncRNA are included (Supplemental Figure S2). PCA analysis was performed; mRNA clustered by time point for 6 h and 7d in NPCs but showed dose differences at 24 h. In contrast, for hepatocytes, there was greater dose and time specific clustering for mRNA (Supplemental Figure S3).

### Radiation injury in the liver quad-culture chip demonstrates similar RNA biomarkers that have previously been published

**Fig. 3:**
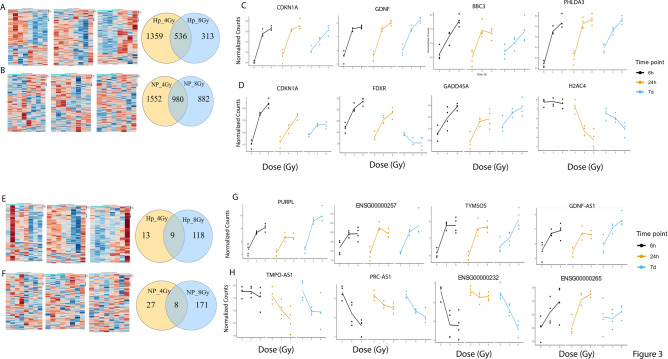
mRNA and lncRNA expression after radiation injury in the liver quad-culture chip shows dose and time dependent changes that may be useful in biomarker discovery. mRNA specific heatmaps for hepatocytes (**A**) and NPCs (**B**) are shown in a time specific manner (left) 6 h, (middle) 24 h and (right) 7d. Venn diagrams showing dose specific mRNA alterations at any time point are shown with 4 Gy as yellow and 8 Gy as blue. (**C**) Altered mRNA in hepatocytes that have been previously identified in our lab and others as potential biomarkers. (**D**) Altered mRNA in NPCs that have been previously identified in our lab and others. lncRNA specific heatmaps for hepatocytes (**E**) and NPCs (**F**) are shown in a time specific manner (left) 6 h, (middle) 24 h and (right) 7d. Venn diagrams showing dose specific lncRNA alterations at any time point are shown with 4 Gy as yellow and 8 Gy as blue. (**G**) Altered lncRNA in hepatocytes that have been previously identified in our lab as potential biomarkers. (**H**) Altered lncRNA in NPCs that have been previously identified in our lab.

Figure 3A,B depict mRNA heatmaps at 4 Gy and 8 Gy for each time point: 6 h (left), 24 h (middle) and 7d (right) for hepatocytes (A) and NPCs (B) that are normalized to their time respective controls (0 Gy). Notably, for hepatocytes at 6 h there is a general upregulation of gene expression after 4 Gy and a downregulation after 8 Gy, in contrast to Day 7 where there is a general upregulation after 8 Gy and a downregulation after 4 Gy. For hepatocytes, 536 mRNA show altered regulation at 4 Gy and 8 Gy compared to controls (Fig. 3A), while 980 mRNA show altered regulation across both doses in NPCs (Fig. 3B). Figure 3C,D shows individual gene plots for mRNA that have previously been associated with radiation injury^19,28,31,32^. Figure 3C are plots based on hepatocyte data while Fig. 3D shows NPCs data. CDKN1A is upregulated across all time points after radiation in NPCs and hepatocytes. Notably, numerous histone genes show decreased expression in NPCs, as we previously observed in our LSECs data from our co-culture model (Fig. 3D, Supplementary Figure S4, Supplementary Table S2,^28^). Interestingly, we also observe decreased expression of three histone genes in hepatocytes as well: H2BC5, H3C8, and H2BC10.

Figure 3E depicts heatmaps for hepatocyte lncRNA and Fig. 3F shows heatmaps for NPCs lncRNA. Most of these hepatocyte gene expression alterations are dose-specific where 118 lncRNA are only altered in 8 Gy samples while 13 are modified at 4 Gy and 9 show alterations at both doses (Fig. 3E). Similarly, NPCs show dose specific changes with 171 lncRNA showing altered regulation only at 8 Gy compared to control and 27 altered only between 4 Gy and control (Fig. 3F). PURPL, TYMSOS and GDNF-AS1 all previously showed altered expression in our liver co-culture hepatocytes and show altered regulation in our quad-culture (Fig. 3G,^28^). TMPO-AS1 and PRC-AS1 showed altered regulation in LSECs in our co-culture and show altered regulation in the NPCs in quad-culture (Fig. 3H,^28^) However, we note other altered mRNA and lncRNA in this quad culture as well (adjusted p-value < 0.05) (Supplemental Table S1, S2). Significantly altered miRNA are also presented; these include miR-34a-5p, a known radiation marker^33,34^, and miR-432-5p, a marker of alcoholic steatohepatitis in a mouse model^35^ (Supplemental Figure S5). Both miRNAs show upregulation; miR-34a-5p is upregulated in hepatocytes in 8 Gy samples at 7d, while miR-432-5p is upregulated in NPCs in 4 Gy and 8 Gy samples at 7d.

### Radiation induces gene changes which may lead to cell death, senescence, fibrosis, inflammation or immune response

**Fig. 4:**
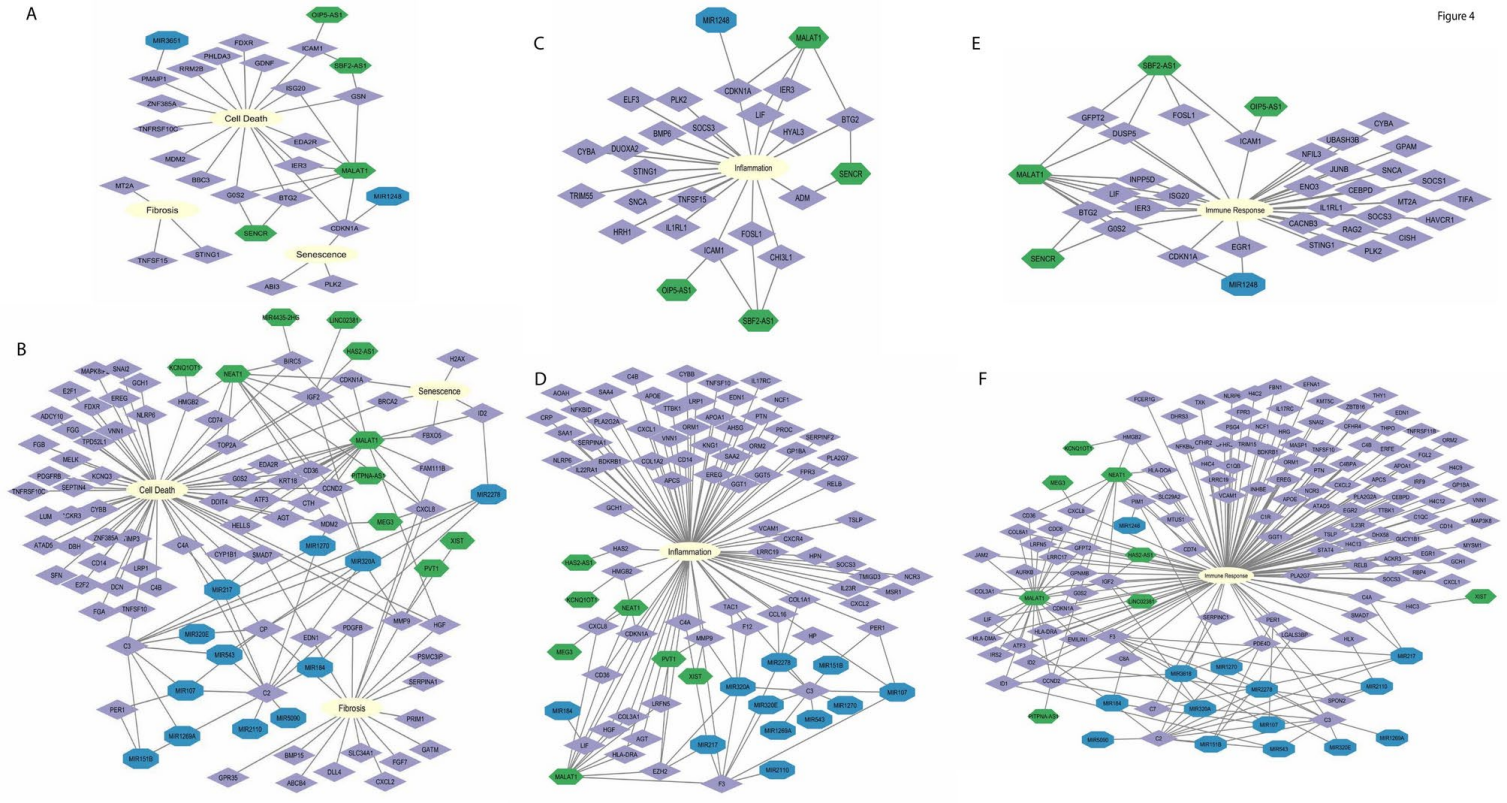
Gene expression changes in our data indicate alterations to cell death, senescence, fibrosis, inflammation and immune response pathways. Networks for radiation injury in hepatocytes (**A**, **C**, **E**) and NPCs (**B**, **D**, **F**) were built with our statistically significant samples (|log2FC|> 1, adjusted p-value ≤ 0.05) across all doses and times. These networks include gene expression changes to genes relevant to fibrosis, cell death and senescence for hepatocytes (**A**) and NPCs (**B**). Network visualizations for inflammation are shown for hepatocytes (**C**) and NPCs (**D**). Network visualizations for immune response are shown for hepatocytes (**E**) and NPCs (**F**). Purple indicates mRNA, green indicates lncRNA, blue indicates miRNA. Network visualizations were simplified for ease of viewability and do not show interactions between mRNAs.

**Fig. 5:**
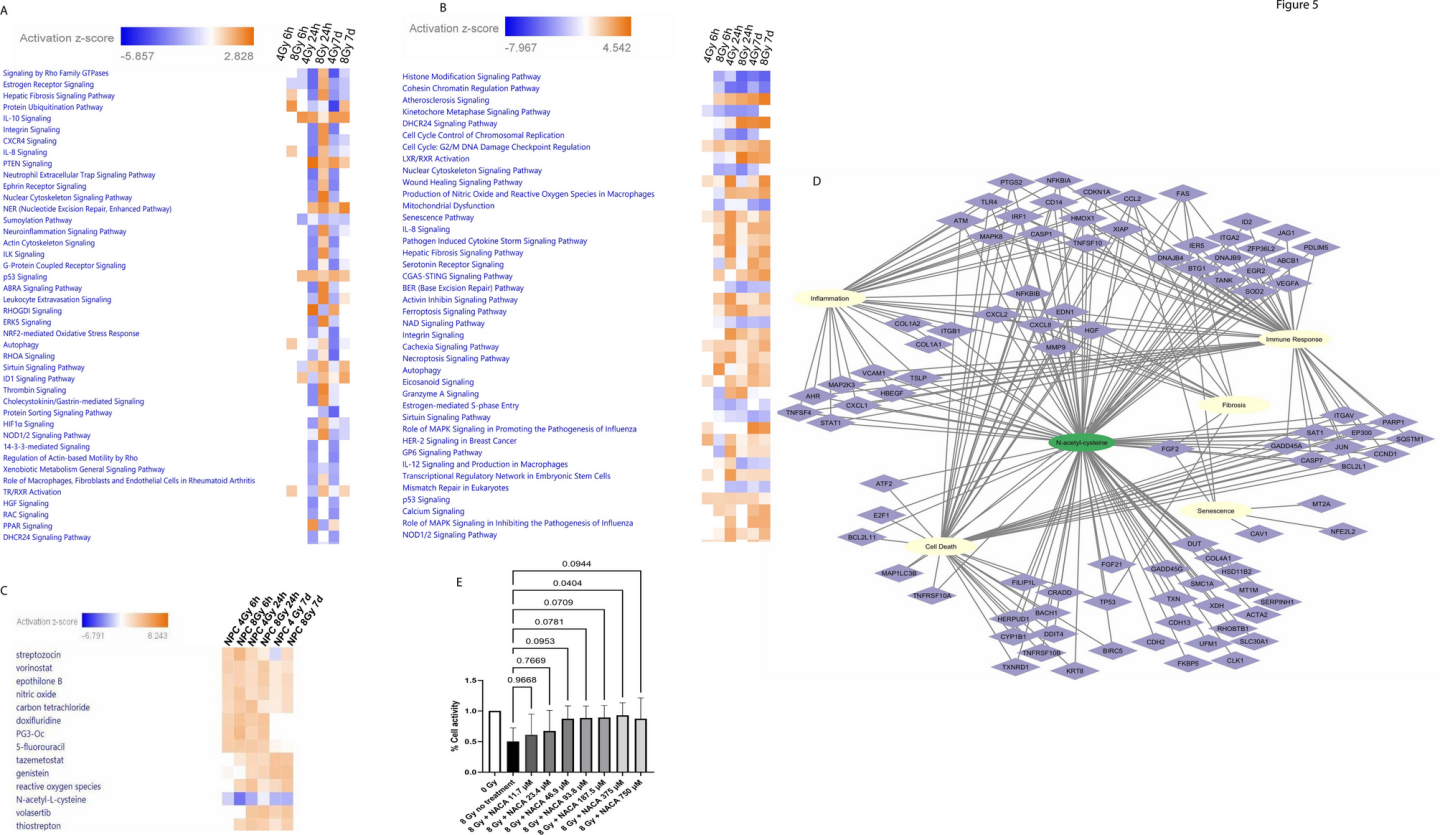
Ingenuity Pathway Analysis predicts activation of cell death pathways in NPCs that may be protected by NAC or NACA. Canonical pathway analysis was performed with IPA using genes where adjusted p-value < 0.05 was used to filter genes. Orange indicates predicted activation of a pathway while blue indicates predicted inhibition. (**A**) Pathways for hepatocytes while (**B**) shows pathways for NPCs. Notably, ferroptosis, necroptosis and autophagy show anticipated activation in NPCs. (**C**) depicts IPA’s upstream regulator drug pathway for NPCs; this indicates potential drugs which would induce similar gene responses. Notably, *N*-acetyl cysteine (NAC) is in blue, suggesting genes which are relevant to this drug pathway are downregulated. (**D**) A network of genes and pathways which are predicted to be modulated by NAC for hepatocytes and NPCs respectively. (**E**) Features a CCK8 cytotoxicity assay 24 h after 8 Gy radiation in LSECs in two-dimensional monolayer culture, n = 6 for each condition, one-way ANOVA with Dunnett’s test, significance at p < 0.05, F = 2.246, DF = 7. Based on this assay, 375 μM of NACA was chosen for the rest of the experiments.

Network diagrams for hepatocytes (Fig. 4A,C,E) and NPCs (Fig. 5B,D,F) are shown. These depict significantly altered genes ([logFC] > 1, p < 0.05) where purple indicates mRNA, green indicates lncRNA and blue indicates miRNA. These diagrams are simplified for ease of viewability and focus on genes which are altered across multiple time points; they do not show interactions between genes. Figures 4A,B show genes altered by radiation injury that act on senescence, fibrosis or cell death pathways. We include heatmaps for the fibrosis pathway for hepatocytes and NPCs with specific interest in genes upregulated at 7d after 8 Gy (Supplemental Figure S6). We further include diagrams for genes relevant to inflammation (Fig. 4C hepatocytes, and Fig. 4D NPCs) and for genes relevant to immune response (Fig. 4E hepatocytes, and Fig. 4F NPCs). These pathways are anticipated to be activated after radiation injury and highlight the potential for the liver-quad culture to recapitulate normal liver response. Notably, CDKN1A, FDXR, MDM2, and EDA2R, genes that are associated with senescence and cell death, show alterations in both hepatocytes and NPCs. CDKN1A is important for all three networks: immune response, cell stress and inflammation. ICAM1 is also altered in hepatocytes across all networks and its protein expression is increased after radiation in NPCs (Fig. 7).

### Ingenuity pathway analysis predicts activation of cell death pathways in NPCs that may be protected by NAC or NACA

As previously noted, radiation induces direct DNA damage and production of reactive oxygen species which can lead to cell death, senescence, mutation or repair and survival. Herein, we show the impact of 4 Gy and 8 Gy radiation (normalized to time matched controls) across 6 h, 24 h and 7d on gene expression pathways using IPA. Figure 5A depicts differences in pathway regulation after radiation injury in hepatocytes and Fig. 5B shows NPCs. Consistent with previous reports, activation of p53 signaling, which plays a central role in gene expression response to radiation, was observed at all time points in both hepatocytes and NPCs^36^. Of particular interest, we observe anticipated activation of Ferroptosis, Necroptosis, and Autophagy in NPCs. We also observed anticipated activation of the Senescence pathway and the Hepatic fibrosis signaling pathway in NPCs. While the Hepatic fibrosis signaling pathway was initially activated at 6 h in 4 Gy and 24 h after 8 Gy samples in hepatocytes, IPA indicated that fibrosis signaling showed anticipated inhibition at later time points. We could observe signaling pathways that were not detected in the liver co-culture chip such as Signaling for production of nitric oxide and Reactive oxygen species in macrophages as well as Pathogen induced cytokine storm. We include data demonstrating difference in pathway activation and inhibition between our liver quad-culture and liver co-culture (Supplemental Figure S7). We also include gene changes relevant to DNA repair pathways for hepatocytes (Supplemental Figure S8) and NPCs (Supplemental Figure S9) as success or failure to repair DNA impacts the cells decision to survive, die or senesce. Noticeably, NPCs show downregulation of DNA repair genes including: RFC2, RFC3, RFC4, FEN1 as well as genes in the nucleotide metabolism pathway including RRM2, and TK1.

We used IPA’s upstream analysis to understand how potential drugs would alter gene expression of our cells once they had been irradiated. Of interest, it appeared that *N*-acetylcysteine (NAC) would inhibit the pathways altered by radiation injury (Fig. 5C). Figure 5D shows the genes altered in our samples which are anticipated to be altered by NAC in NPCs; these genes are known to modulate pathways involved in cell death, fibrosis and senescence. NACA is a modified molecule of NAC with greater cellular permeability. NACA is able to replenish glutathione levels and decrease lipid peroxidation in response to oxidative stress^37,38^. Data from our work and others indicates LSECs may be the most sensitive cell type in the liver^28,39^. We then studied the ability of NACA to protect LSECs in two-dimensional monolayer culture; cells received 8 Gy radiation, and the CCK8 cell viability assay was performed (Fig. 5E). 8 Gy radiation reduced cell viability by approximately 50%, while pre-incubation with 375 μM of NACA was observed to significantly improve LSECs viability. Therefore, we chose 375 μM of NACA as a candidate for therapeutic countermeasures against RILD.

### NACA protects cells from DNA damage and decreases markers of cell stress and senescence

**Fig. 6:**
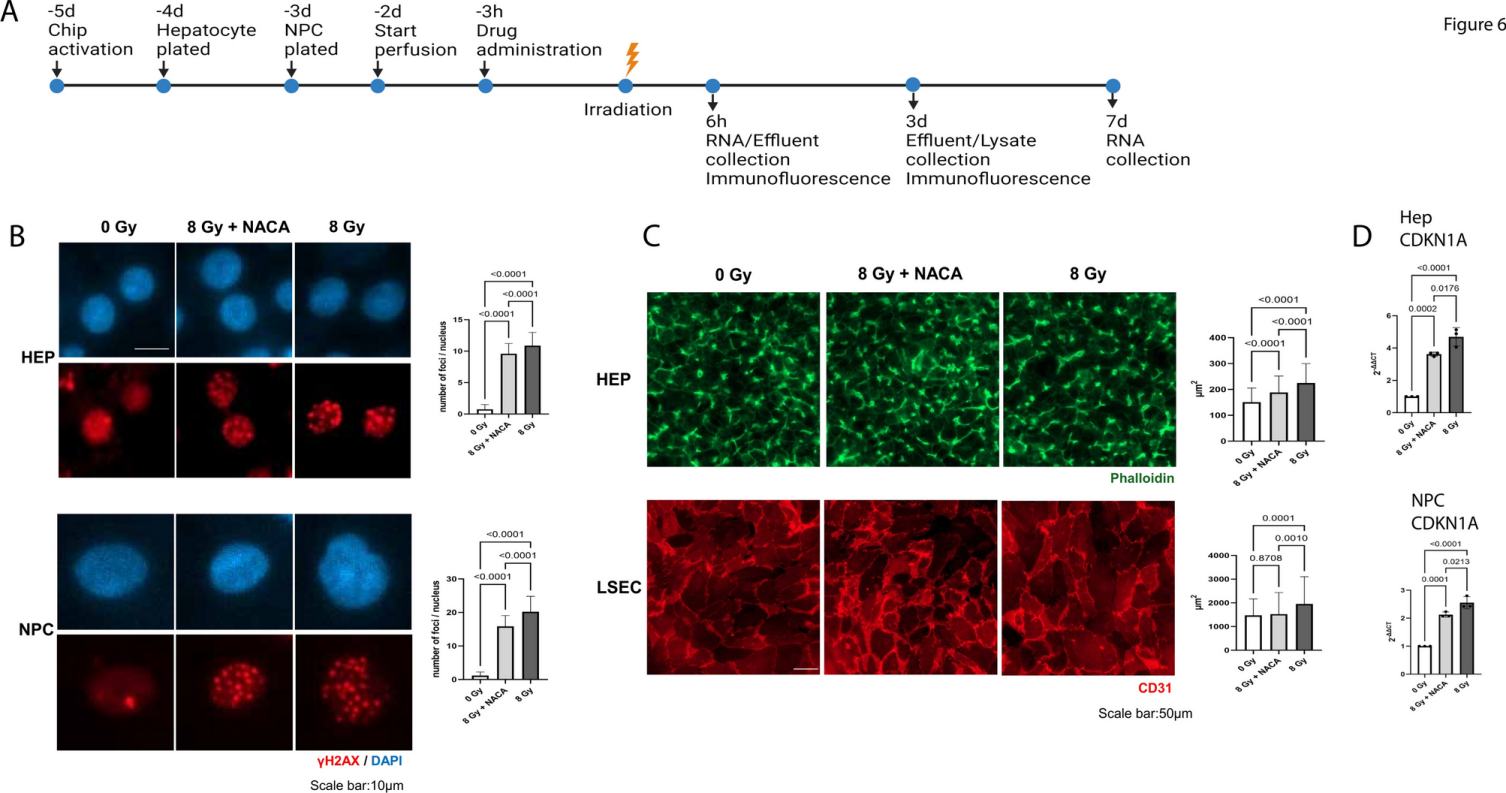
NACA decreases DNA damage, cells stress and senescence. (**A**) shows a timeline of events from initial chip activation to cell plating on liver-chip, to pre-incubation with NACA, to radiation injury and then the timepoint for collection (6 h, 3d, 7d). Note that liver-chips were pre-incubated with NACA (375 μM) for 3 h before radiation injury for all experiments. (**B**) γ-H2AX staining for hepatocytes and NPCs at 0 Gy (left), 8 Gy + NACA (middle) and 8 Gy (right) samples. Foci counts were performed for hepatocytes and NPCs, then compared to total nuclei. For γ-H2AX in hepatocytes a total of 102 nuclei were analyzed in 3 chips, one-way ANOVA with Tukey’s test was performed, significance at p < 0.05, F = 1206, DF = 2. For NPCs n = 108 nuclei analyzed in 3 chips, one-way ANOVA with Tukey’s test performed, significance at p < 0.05, F = 983.4, DF = 2. (**C**) Phalloidin staining of hepatocytes and CD31 staining in LSECs as a method to measure cell area 6 h post sham, 8 Gy + NACA or 8 Gy radiation. For hepatocytes 3 chips were measured, (0 Gy n = 135, 8 Gy + NACA n = 114, 8 Gy n = 104), one-way ANOVA with Tukey’s test, significance at p < 0.05, F = 39.31, DF = 2. For LSECs 3 chips were measured (0 Gy n = 146, 8 Gy + NACA n = 133, 8 Gy n = 111), one-way ANOVA with Tukey’s test, significance at p < 0.05, F = 9.89, DF = 2. (**D**) shows CDKN1A RT-PCR for hepatocytes (upper) and NPCs (lower) 6 h after radiation, n = 3, one-way ANOVA with Tukey’s test was used, significance at p < 0.05, F(HEP) = 97.70, F(NPCs) = 103.1, DF = 2. Sham are shown in white, 8 Gy irradiated are in black and 8 Gy + NACA are in gray.

For the subsequent experiments, our timeline is noted in Fig. 6A wherein cells received 375 μM of NACA 3 h prior to radiation injury. Then lysate and effluent collection occurred 6 h, 3d or 7d post injury. We preincubated hepatocytes and NPCs with NACA prior to 8 Gy radiation then performed γ-H2AX staining 6 h post radiation. Figure 6B shows γ-H2AX, a known indicator of DNA double strand breaks, in hepatocytes and NPCs 6 h post radiation after sham (0 Gy), 8 Gy or 8 Gy + NACA. Foci per nucleus are shown for hepatocytes and NPCs. Cell surface area was measured with phalloidin for hepatocytes and CD31 for LSECs 6 h post radiation injury with or without NACA (Fig. 6B). CD31 is a known marker of endothelial cells (and is not observed in Kupffer cells or HSCs) and was previously used to show increased cell surface size in human umbilical vein endothelial cells after radiation injury^40,41^. Similarly, hepatocytes have shown an increased cell surface area in response to stress and potential attempts at regeneration^42^. We note that at 6 h post radiation, both hepatocytes and LSECs are showing signs of stress marked by increases in cell size (Fig. 6C). Notably 8 Gy + NACA decreases cell surface area in LSECs and hepatocytes. We also observe upregulation of the senescence marker CDKN1A at 6 h in hepatocytes and NPCs (Fig. 6D). This increase is prevented by the pre-incubation of cells with NACA. Thus, CDKN1A may be useful in determining RILD progression and predicting patient response to RILD treatments. Other markers of DNA damage and radiation response including PCNA, MDM2, DINO and the senescence marker LMNB1 are shown after 8 Gy radiation or 8 Gy + NACA (Supplemental Figure S10).

### NACA protects NPCs from cell death, endothelial dysfunction, inflammation, and HSCs activation, and hepatocytes from metabolic dysfunction

**Fig. 7:**
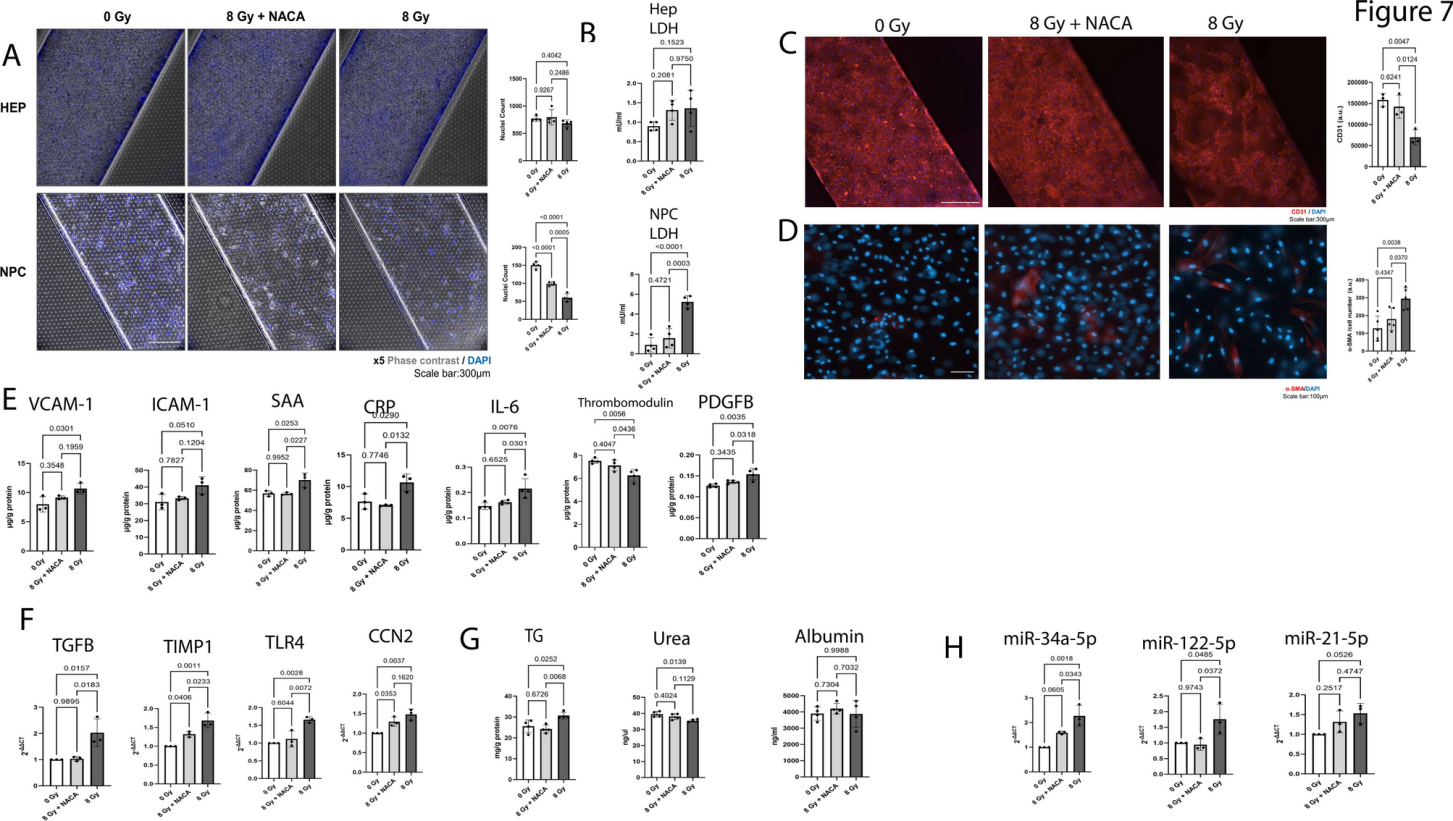
NACA improves cell survival in NPCs and ameliorates pathophysiological changes related to RILD. Radiation induced cell death and functional markers were measured at 3 days or 7 days post radiation injury. (**A**) Phase contrast image with DAPI staining to determine cell death at 3 days post 8 Gy radiation, hepatocytes (upper) and NPCs (lower). Briefly, nuclei were counted 3 days after radiation in 4 areas across 4 chips, one-way ANOVA with Tukey’s test, significance at p < 0.05, F(HEP) = 1.647, F(NPCs) = 102.1, DF = 2. (**B**) LDH effluent measured by ELISA for hepatocytes (left) and NPCs (right). One-way ANOVA with Tukey’s test was performed, significance at p < 0.05, F(HEP) = 2.58, F(NPCs) = 35.52, DF = 2. (**C**) CD31 is decreased in irradiated samples compared to 8 Gy + NACA, 3 days after radiation. CD31 fluorescence was measured across 3 areas in 3 chips, one-way ANOVA with Tukey’s test, significance at p < 0.05, F = 15.56, DF = 2. (**D**) α-SMA is increased in irradiated samples compared to 8 Gy + NACA + radiation. 3 days after radiation α-SMA was measured in 5 areas across 3 chips and normalized to cell number (DAPI), one-way ANOVA with Tukey’s test, significance at p < 0.05, F = 8.867, DF = 2. (**E**) depicts ELISA assays that were performed using NPCs lysates 3 days post radiation injury. These included VCAM-1, ICAM-1, SAA and CRP which were measured in 3 chips, as well as IL-6, Thrombomodulin, PDGFB, level were measured in 4 chips. One-way ANOVA with Tukey’s test was performed for all ELISAs, significance at p < 0.05, F(VCAM-1) = 6.128, F(ICAM-1) = 5.132, F(SAA) = 9.053, F(CRP) = 10.22, F(IL-6) = 8.9, F(Thrombomodulin) = 9.339, F(PDGFB) = 10.84, DF = 2. (**F**) show RT-PCR validation of TGFB1, TIMP1, TLR4 and CCN2 mRNA levels in NPCs 3 days after radiation, n = 3, one-way ANOVA with Tukey’s test, significance at p < 0.05, F(TGFB1) = 10.61, F(TIMP1) = 24.16, F(TLR4) = 19.19, F(CCN2) = 15.33, DF = 2. (**G**) depicts ELISA results from hepatocytes. Briefly TG, Urea and albumin were measured 3 days after radiation injury. TG utilized cell lysate while the Urea and Albumin assays relied on effluent. Results are from 4 chips, one-way ANOVA with Tukey’s test, significance at p < 0.05, F (TG) = 8.625, F(Urea) = 6.692, F(Alb) = 0.45, DF = 2. (**G**) miRNA RT-PCR for hepatocytes relevant to radiation damage. RT-PCR was performed 7 days post radiation injury, n = 3, ANOVA with Tukey’s test, F(miR-34a-5p) = 19.83, F(miR-122-5p) = 6.859, F(miR-21-5p) = 4.634, DF = 2.

Three days after radiation injury we used phase contrast microscopy to assess cell death in our liver quad-culture chip and observed a significant reduction in cell numbers in the NPCs channel; there was no significant radiation induced cell death in hepatocytes (Fig. 7A). We observed increases in cell death as measured by LDH effluent in NPCs after 8 Gy radiation that is partially protected by pre-incubation with NACA (Fig. 7B). However, hepatocytes do not show increases in LDH effluent. We then performed RT-PCR on key genes in the ferroptosis pathway^43,44^ focusing only on NPCs because we did not see significant cell death in hepatocytes. At 6 h and 3 days post-irradiation, several ferroptosis markers were affected in the NPCs, including downregulation of GPX4, HSBP1, FANCD2, and upregulation of PTGS2, ACSL4, TFRC and SQLE (Supplemental Figure S11). We validated expression of miR-432-5p which is associated with ferroptosis as well as steatohepatitis^35^ and note that it shows increased expression, as was observed in our sequencing data (Supplemental Figure S5). These findings suggest that ferroptosis pathways may be active and involved in the cell death of NPCs. Ferroptosis pathway activation was observed in LSECs in our co-culture as well^28^. We sought to determine if most cell death was due to LSECs, so we used CD31 and DAPI to highlight LSECs presence in the chip (Fig. 7C). We observed decreased CD31 expression compared to DAPI. We then observed increases in alpha smooth muscle actin (α-SMA) 3 days after radiation injury in NPCs (Fig. 7D); this is a marker of HSCs activation, endothelial to mesenchymal transition (EMT) and potentially fibrosis^45^. We note that NACA decreases relative α-SMA expression in NPCs compared to irradiated NPCs alone. We further observed increased expression of VCAM-1, ICAM-1, SAA, CRP, IL-6, thrombomodulin, PDGF in NPCs cell lysates (Fig. 7E). These are markers of endothelial injury and inflammation^46^ that were increased after 8 Gy exposure but were decreased after pre-incubation with NACA. We validated upregulation of TGF-B, TIMP1, TLR4, and CCN2, markers of HSCs activation^47^, in NPCs after radiation alone. This upregulation was less notable when cells were pre-incubated with NACA prior to exposure (Fig. 7F).

Interestingly, we observed other pathway changes in hepatocytes that were not predicted by software (IPA or R databases) but may be useful markers for radiation injury. We observed changes in triglyceride (TG) levels in hepatocyte lysate (Fig. 7G) as well as decreases in Urea at 3 days post injury but no changes to albumin from hepatocyte effluent. NACA incubation decreased TG in pre-treated cells compared to radiation alone cells. Similarly, known miRNA markers of Metabolic-associated Fatty Liver Disease^48^ including miR-34a-5p, miR-21-5p and miR-122-5p were upregulated with radiation injury (Fig. 7H).

## Discussion

**Fig. 8:**
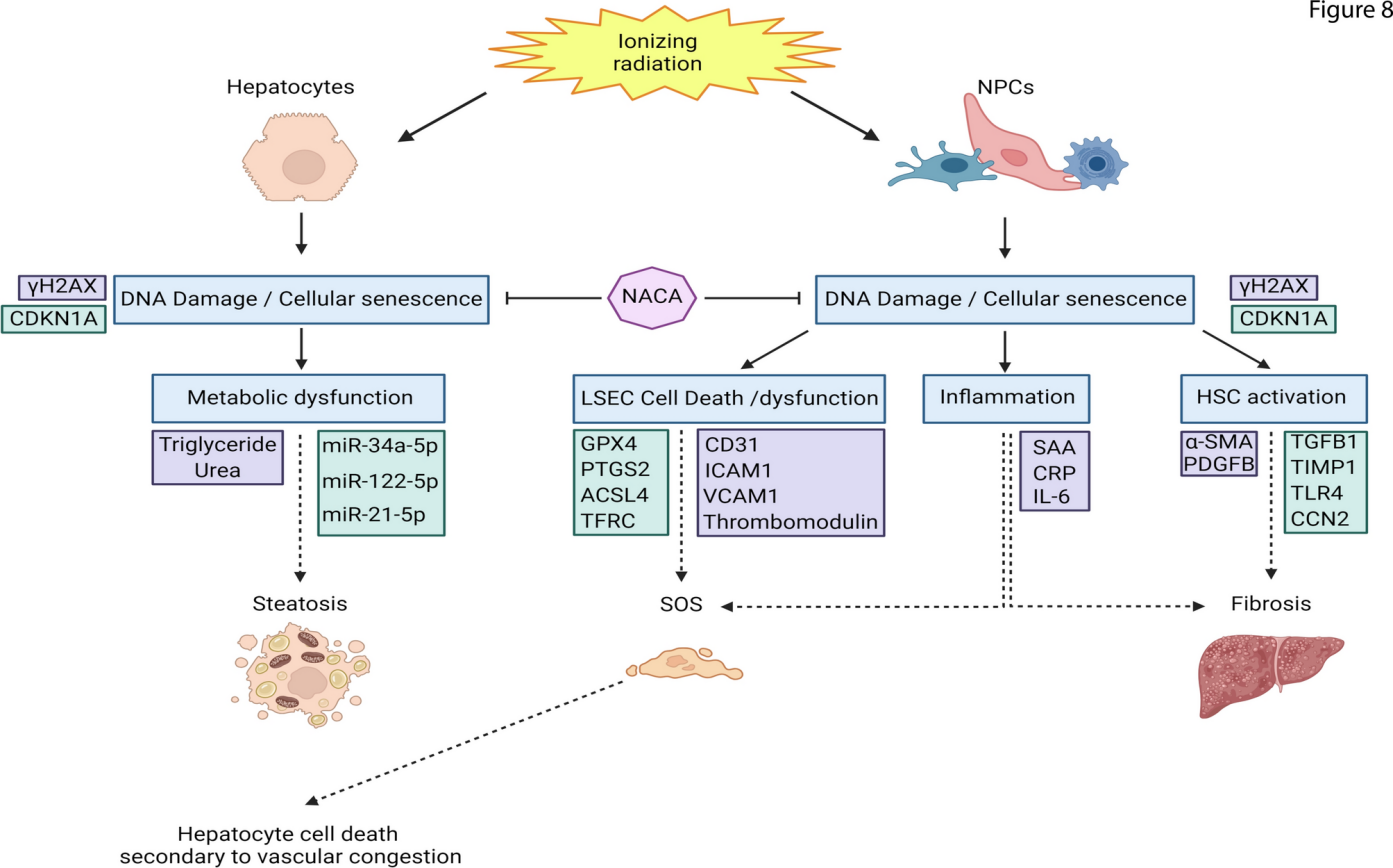
Liver quad-culture chip model is an effective model for investigating the microenvironment in RILD and for evaluating the efficacy of therapeutic countermeasures and biomarkers. Radiation exposure to the chip model resulted in DNA damage and cellular senescence of hepatocytes and NPCs. This led to metabolic dysfunction in hepatocytes and led to LSECs cell death, endothelial dysfunction, inflammation, and HSCs activation in NPCs. NACA exhibited the capacity to mitigate DNA damage and cellular senescence and other pathophysiological changes including triglyceride accumulation. We predict these NACA induced changes would help to inhibit steatosis, sinusoidal obstruction syndrome and liver fibrosis in NPCs. Purple refers to protein dysregulation validated by ELISA assays and immunofluorescence while green refers to RNA dysregulation validated by RT-PCR.

In this study, we highlight the impact of radiation injury on the two cell groups: hepatocytes and NPCs and the ability of NACA to suppress these pathways and protect the cells. NACA was able to reduce DNA damage and cellular senescence found in both cell types compared to irradiated samples. NACA reduced triglyceride accumulation in hepatocytes after radiation, which may have implications for steatosis in the liver. Moreover, NACA reduced LSECs cell death and inflammation as well as HSCs activation after radiation injury in NPCs. Figure 8 depicts the observed impact of NACA on radiation response in the liver quad-culture model. This may have an impact on SOS and RILD progression. CDKN1A and miR-34a-5p as RNA markers of radiation injury markers were validated in our quad-culture and may be utilized as markers of RILD.

DNA damage and cellular senescence of hepatocytes and NPCs induce various biological changes such as metabolic dysfunction, inflammation, endothelial dysfunction and HSCs activation in the liver^49^. In hepatocytes, we detected increased expression of triglycerides and the upregulation of microRNAs that are associated with steatosis development: miR-34a, miR-122 and miR-21^48^. In NPCs, we detected the increased expression of leucocyte adhesion proteins including ICAM-1 and VCAM-1, increased expression of the inflammatory cytokine IL-6, increased expression of acute phase proteins including CRP and SAA. These are known to be associated with inflammation and endothelial dysfunction. We also detected the decreased expression of thrombomodulin which activates platelets and may create a prothrombotic state that leads to SOS^50^. Furthermore, we detected an increase of PDGFB which is a potent factor associated with early HSCs activation; these activated HSCs express high levels of α-SMA, TGF-B, TIMP1, TLR4, and CCN2^51^ . We could detect these metabolic dysfunction, inflammation, endothelial dysfunction, HSCs activation in irradiated samples. NACA was able to alleviate the impact of radiation on these markers, and potentially the biological changes associated with them. Taken together, this suggests the liver quad-culture chip is an effective model not only for studying RILD, as it mimics the biological changes and microenvironment in RILD but also for testing potential therapeutic countermeasures.

CDKN1A has been reported as a biomarker of radiation damage response in cancer patients undergoing total body irradiation^52^. miR-34a-5p has also previously been reported as a bio-dosimetry gene of irradiation in a murine model; it regulates oxidative stress, DNA damage, cell cycle and cellular senescence by regulating p53 in RILD^19,53^. We validated the dose-dependent upregulation of CDKN1A expression in both hepatocytes and NPCs and upregulation of miR-34a-5p expression in hepatocytes. NACA decreased the expressions of CDKN1A and miR34a-5p in the liver quad-culture chip compared to untreated irradiated cells. CDKN1A and miR-34a-5p may serve as useful biomarkers for bio-dosimetry and treatment efficacy in RILD. While liver biopsy is needed to identify these RNA dysregulations in clinical practice, recent studies have focused on serum non-coding RNAs as non-invasive biomarkers for liver fibrosis, including miR-34a^54^. Additionally, research on measuring microRNA in chip effluent^55^ suggests the potential for using circulating non-coding RNAs as biomarkers for RILD. This indicates that the liver quad-culture chip could be a promising model for studying RNA biomarkers.

Our findings highlight important differences related to genes involved in cell death, senescence and fibrosis between NPCs and hepatocytes following radiation exposure. A comprehensive understanding of how NPCs and hepatocytes react to radiation injury can lead to better mitigation strategies and treatment options. Despite the same doses of radiation (4 Gy or 8 Gy) being administered to both cell types there were differences in gene expression alteration. Only NPCs show downregulation of MKI67, MYBL2, and LMNB1, repression of these markers is associated with activation of the senescence pathway^56^ . In contrast, hepatocytes showed altered regulation of genes relevant to DNA metabolism including: RRM2B, TYMS, and genes relevant to immune function including: HES2, IGSF1, STING1, and INPP5D. Differences in gene expression were observed between the lncRNA altered by radiation. In hepatocytes there was altered regulation of PURPL, LETR1, LIMASI, and SENCR. Both PURPL and SENCR were upregulated in hepatocytes; research on their roles in normal tissues is limited, although they are linked to proliferation and drug resistance in cancers^57^. SENCR is also associated with proliferation and angiogenesis in human embryonic stem cells through modulation of multiple genes^58^. While LETR1 and LIMASI are associated with immune response. Interestingly in NPCs, XIST is downregulated, since this is a known regulator of gene silencing^59^, this may be relevant to the observed decreases in histone expression that we observe most notably in NPCs. MALAT1 is altered in both NPCs and hepatocytes but it is downregulated at 24 h in hepatocytes and upregulated in NPCs.

Our findings confirmed a significant increase in cell death in NPCs after irradiation with 8 Gy radiation. Although, no increase was observed in hepatocytes. The difference in DNA damage repair and cellular senescence between NPCs and hepatocytes may influence this known difference in radiosensitivity^53^. We have also previously compared gene expression biomarkers between mouse liver and human liver co-culture chip^28^. In the animal model, it is difficult to analyze RNA dysregulation in hepatocytes and NPCs separately, and it may be difficult to detect RNA dysregulation in NPCs with a smaller number of the cells in the liver. The liver quad-culture chip can clearly distinguish NPCs from hepatocytes. This may be an ideal model to analyze the mechanism of liver disease in which NPCs play a major role, such as SOS and fibrosis.

We have previously investigated radiation injury using the liver co-culture chip which consisted only of hepatocytes and LSECs^28^. Although we were able to detect RNA dysregulation such as activation of the ferroptosis signaling pathway in LSECs, we were unable to detect morphological changes such as LSEC death, biological changes such as inflammation, and fibrosis. We compared the NPCs from our quad-culture to the LSECs in the co-culture to examine how the addition of Kupffer cells and HSCs impacted radiation response. Along with other pathway changes, the senescence pathway was more profoundly activated in the quad-culture in comparison to the co-culture. Also, the hepatic fibrosis signaling, and the pathogen induced cytokine storm pathways were detected only in the quad-culture using IPA (Supplemental Figure S7). Furthermore, we observed cell death in the liver quad-culture, especially in LSECs; this might be due to presence of Kupffer cells as previously suggested^13^. However, follow-up studies to determine mechanism of Kupffer cell and HSC induced death or damage of LSECs is necessary.

We have discussed the effectiveness of the liver quad-culture chip, some limitations remain. This liver quad-chip model cannot predict how RILD effects other organs of the body, nor can it predict how other organs might influence RILD. Additionally, while hepatocytes and Kupffer cells may interact in the human liver, a porous barrier separates NPCs from hepatocytes in our liver quad-culture (Fig. 1). We acknowledge this limitation and note that another study comparing transcriptomics and proteomics from patients with hepatic steatosis and a liver-chip model demonstrated that the chip successfully recapitulated key findings from human patients^60^. While the liver on a chip is not a perfect model, it can accurately represent aspects of the liver’s response to injury.

Our model lacks the white blood cells, red blood cells, platelets, and immune cells that are present in the circulating blood and would play an important role in RILD. Metabolic dysfunction may also be regulated by hormones and cytokines secreted by other organs and tissues, but our model is unable to examine these external factors. To better understand the systemic effects of radiation and potential interactions with other organs, further improvements may be useful, similar to the addition of Kupffer cells and HSCs in this study. For example, perfusion of human whole blood or integration of multi-organ chips could provide more comprehensive insights^61,62^. Another potential concern, long-term experiments are not suitable with normal human cells in the liver-chip due to the limited ability of normal human cells to survive and proliferate. However, in clinical practice, SOS and liver fibrosis are typically reported weeks to years after radiotherapy^22^. In our quad-culture we can only show the impact of a mitigator in the early period. We anticipate this effect indicates long-term protection, but longer-term studies in another model system will be needed to verify this protection against development of clinical symptoms. Another limitation is chemical interactions between a prospective therapeutic drug and the chip material. It has been reported that pharmaceutical drugs with a large molecular size and greater hydrophobicity interact with the chip membrane^63^. We chose NACA for our drug experiment based on its molecular weight and its octanol–water partition coefficient (logP value), a measure of hydrophobicity. We chose NACA after an extensive literature search for therapeutic agents that would be predicted to reduce the radiation-induced LSEC cell death we observed in 2D LSEC cultures. Compared to the other agents, NACA was superior because of its ability to exert its effect at concentrations lower than the maximum plasma concentration that would be expected to be reached after clinical administration^64^.

It is recommended that a model for using drugs with high molecular weight or high logP value should use MPS, which consists of low-drug-absorbing materials. Notably, roughly half of early clinical trials fail because of treatment toxicity, including liver injury; clinical studies suggest that ALT value during an initial drug induced liver injury episode would predict secondary injuries if patients were re-challenged with the drug^65^. Cirrhosis and other diseases of the liver may also be important in determining potential drug toxicity^66^ . Drug studies should be undertaken to determine drug safety in pathologic conditions as well as normal liver tissue as part of a continued effort to improve patient safety before clinical trials. In addition, therapeutic drug experiments should be conducted using concentrations that can actually be administered in clinical practice compared to pharmacokinetic profile.

Addressing the identified limitations and exploring future enhancements could further improve the utility and applicability of this model in both basic research and therapeutic development. Further studies are needed to assess the impact of NACA administered after radiation as a potential treatment. Prior evidence from NAC/NACA studies indicate that these medications protect against neuronal degeneration after traumatic brain injury and liver injury after paracetamol (acetaminophen) overdose^67,68^. Additionally, NAC was also shown to prevent lung function decline in cystic fibrosis patients, as measured by forced expiratory volume in 1 s (FEV1), over a 24-week period^69^. These findings suggest that NAC/NACA could be beneficial for both acute injuries and chronic conditions, raising the potential for their use in mitigating radiation-induced adverse effects in radiotherapy patients, although more evidence is needed to confirm NACA’s efficacy in this context.

In conclusion, we demonstrated that a liver quad-culture chip recapitulated anticipated alterations in the normal human liver after injury including alterations in genes and proteins relevant to cell death, senescence, fibrosis and inflammation. We also validated this model as a method for evaluating potential therapeutic countermeasure like NACA and for evaluating RNA marker. The liver quad-culture chip model could have significant implications for diagnostic and therapeutic strategies aimed at protecting the liver from radiation damage in both clinical settings and scenarios involving radiation exposure.

## Methods

### Liver-on-a-chip quad-culture

Primary human hepatocytes, LSECs, Kupffer cells and hepatic stellate cells were obtained from Emulate Inc. Similarly, all equipment including Chip-S1, Pod module, Zoë Culture Module, and the Orb Hub Module were obtained from Emulate Inc. The Chip-S1 has two channels that are separated by a porous polydimethylsiloxane (PDMS) membrane that allows for cell–cell interaction *However*, these two channels are fluidically independent; this allows for independent dosing of the epithelial or endothelial channel and independent collection of perfused media. The Zoë Culture Module and Orb Hub Module provide dynamic flow of media, which is meant to reproduce the blood flow and sheer pressure in in vivo conditions. The experimental setup for liver quad cultures has been previously described^26,70^*.* Briefly, on day − 7, flasks were coated with Attachment Factor (Cell Systems) and LSECs were plated and expanded. Complete LSEC medium contains Cell Systems medium with final concentrations of 1% Pen/Strep (Sigma), 2% Culture-Boost (Cell Systems), and 10% Fetal Bovine Serum (FBS) (Sigma). On day − 6, empty chips were activated using Emulate proprietary reagents, and both channels were coated with collagen I (Corning) and fibronectin (Thermofisher) to mimic extracellular matrix (ECM) components. On day − 5, hepatocytes were thawed and seeded directly into the epithelial channel of the chips at a density of 3.5 × 10^6^ cells/mL. Complete hepatocyte seeding medium contains Williams’ Medium E (Sigma) with final concentrations of 1% Pen/Strep (Sigma), 1% L-GlutaMAX (Gibco), 1% Insulin-Transferring-Selenium (ITS) (Gibco), 50 µg/mL Ascorbic Acid (Sigma), 1 μM dexamethasone (Sigma), and 5% FBS (Sigma). On day − 4, the hepatocyte Matrigel (Corning) overlay was performed in the epithelial channel. This procedure promotes a three-dimensional matrix for hepatocytes to grow in ECM sandwich culture. The hepatocyte maintenance medium contains Williams’ Medium E (Sigma) with final concentrations of 1% Pen/Strep (Sigma), 1% L-GlutaMAX (Gibco), 1% ITS (Gibco), 50 µg/mL Ascorbic Acid (Sigma), and 100 nM Dexamethasone (Sigma). On day − 3, Kupffer cells and hepatic stellate cells were thawed; LSECs, Kupffer cells and hepatic stellate cells were then seeded on the endothelial channel of the chip with LSECs at a final density range of 4 × 10^6^ cells/mL, Kupffer cells at 2 × 10^6^ cells/mL, hepatic stellate cells at a density of 0.1 × 10^6^ cells/mL. NPC seeding medium contains Williams’ Medium E (Sigma) with final concentrations of 1% Pen/Strep (Sigma), 1% L-GlutaMAX (Gibco), 1% ITS (Gibco), 50 μg/mL Ascorbic Acid (Sigma), and 10% FBS (Sigma). On Day − 2, media was degassed using Steriflip-connected tubes (Millipore) to minimize the formation of bubbles. The Chips were connected to the Pod and then placed in the Zoë to begin fluid flow of media at 30 µL/h . NPC maintenance media was composed of the same components of NPC seeding media, with a reduction of FBS to 2%. Timeline is shown in Fig. 1B.

### Irradiation and liver-chip dosimetry

Cells and liver-chips were irradiated using an X-Rad 320 (Precision X-ray Inc.), which produces 320-kV photons at 12.5 mA. Cells were sham irradiated (0 Gy) or received a single dose of 4 Gy or 8 Gy. One liver-chip at a time was irradiated to limit total time away from temperature- and fluid-controlled conditions. The total time away from the incubator for irradiation was less than 5 min per sample. The flow for all chips was stopped and re-started at the same time. Irradiated cells were collected with time matched sham at 6 h, 24 h, and 7 days (7d) post-radiation for RNA-seq analysis.

To ensure the liver-chips were exposed to the expected doses, we performed radiation dosimetry analyses and established a setup for the chips that differed from traditional in vitro models. The irradiator was calibrated according to the Task Group Report 61 (TG61): AAPM protocol for 40–300 kV x‐ray beam dosimetry in radiotherapy and radiobiology. To reduce low energy radiation scatter from the metal baseplate of the irradiator cabinet, a polystyrene block (25 × 25 × 2 cm) was used both during calibration and experimental irradiation. Because the irradiator field was smaller than the chamber, thermoluminescence dosimeter chips (TLDCs) (3 × 3 × 1 mm) were used to calibrate the chamber to a known dose. To determine dose rate, three TLDCs were irradiated under the TG61 condition. The process was performed twice for a total of six TLDCs irradiated, and the average dose was taken and used for subsequent experiments. The polystyrene block was marked to establish uniform placement of liver-chips each time.

To validate NACA in Liver-on-a-Chip Quad-Culture, NACA (Sigma, A0737) was reconstituted in DPBS (Corning) and added to endothelial channels at concentration 375 μM, 3 h before irradiation procedure.

### RNA isolation

Cell lysis for RNA isolation was performed as outlined by Emulate protocol. Briefly, NPCs are collected from endothelial channel first, followed by hepatocytes from epithelial channel to prevent cross-contamination. Both channels were rinsed with cold DPBS and trypsin–EDTA (Sigma) was added to the endothelial channel to collect NPCs. After transferring cell suspension to a tube, FBS was added to stop trypsinization. Then, add TRIzol Reagents to the cells in the tube. Chips were inspected under microscope to confirm complete collection of NPCs from endothelial channel. For the hepatocytes, add TRIzol Reagents to the epithelial channel directly and transferring cell lysate to a tube. The cell lysates were stored at − 80 °C for downstream RNA extraction. Subsequent purification was performed as outlined by TRIzol RNA Isolation Reagents and the PureLink Mini kit (Invitrogen).

### RNA-seq data processing

Raw reads from the RNA-seq data were first filtered to remove reads with > 10% uncertain nucleotides and/or > 50% proportion of low-quality (i.e., < 20) bases. Reads with adapter contamination were also removed. After quality filtering, hierarchical indexing for spliced alignment of transcripts (HISAT2) was used for mapping reads to the human reference genome^71^. Featurecounts, a highly efficient read summarization method, was then implemented to assign reads to both protein coding and long non-coding genomic features^72^.

### SMARTer Stranded V2

RNA sample quality was assessed by BioAnalyzer High Sensitivity RNA Assay (Agilent Technologies Inc., California, USA) and quantified by AccuBlue® Broad Range RNA Quantitation assay (Biotium, California, USA). Library construction is performed based on manufacturer’s recommendation for SMARTer® Stranded Total RNA-Seq Kit v2—Pico Input RNA Kit (Takara Bio USA Inc., California, USA). Final library quantity was measured by KAPA SYBR® FAST qPCR and library quality evaluated by TapeStation HSD1000 ScreenTape (Agilent Technologies, CA, USA). Average final library size is 450 bp and Illumina® 8-nt dual-indices were used. Equimolar pooling of libraries was performed based on QC values and sequenced on an Illumina® NovaSeq 6000 (Illumina, California, USA) with a read length configuration of 150 PE for 40 M PE reads per sample (20 M in each direction).

### QIAseq miRNA library preparation

Sample quality was assessed by BioAnalyzer High Sensitivity RNA Assay (Agilent Technologies Inc., California, USA) and quantified by AccuBlue® Broad Range RNA Quantitation assay (Biotium, California, USA). Library preparation was performed with QIAseq miRNA Library Preparation Kit (QIAGEN, Hilden, Germany) following manufacturer’s instructions. Average final library size is 200 bp. Illumina 10-nt unique dual-indices were used for multiplexing. Samples were pooled and sequenced on Illumina NovaSeq 6000 sequencer for 150 bp read length in paired-end mode, with an output of 20 million paired end reads per sample (10 M in each direction). The data was ultimately trimmed to 1 × 50 prior to data delivery.

### RNA-seq data processing

Read quality and adapter contamination were assessed using the FastQC (https://github.com/s-andrews/FastQC, v0.11.9) and low-quality reads were removed using the read trimming tool Trimmomatic (v0.39)^73^. After read filtering, the STAR (v2.7.10a)^74^ aligner was used for mapping reads to the human reference genome (primary genome assembly GRCh38) and assigning read counts to mRNA, miRNA, and lncRNA genomic features.

### RNA-sequencing data analysis

Differential gene expression analyses were performed on raw read counts. All pair-wise comparisons for differential gene expression analyses were conducted using the DESeq2 R package^75^, and included comparisons between radiation treatment dose levels (4 Gy, and 8 Gy) and control (0 Gy) across three dosage timepoints (6 h, 24 h, 7d). Genes with an adjusted p-value (FDR) < 0.05 were considered differentially expressed and utilized in downstream analysis. Where noted, more stringent log fold-change thresholds were employed in addition to adjusted p-value filtering.

### Gene set enrichment analysis

Enrichment analysis was conducted using the Generally Applicable Gene-set Enrichment (GAGE) (implemented through the R package gage)^76^, which is a parametric gene randomization procedure that utilizes log-based fold changes to robustly assess significantly regulated pathways across pairwise sample comparisons. For each pairwise sample comparison, the log2 fold-change values output by DESeq2 for every gene were input into the gage package and enrichment for pathways from the Kyoto Encyclopedia of Genes and Genomes (KEGG) database were assessed. Pathways with a q-value ≤ 0.2 were considered for downstream analysis. Ingenuity Pathway Analysis (IPA) (v127006219) was also performed using statistically significant genes to determine pathway changes (|log2FC|> 1, adjusted p-value < 0.05).

### Biological network analysis and visualization

Interactions between mRNA miRNA and lncRNA were downloaded from the miRDB^77^ and LncSEA^78^ databases, respectively. Only coding and non-coding RNAs that were differentially expressed (i.e., adjusted p-value ≤ 0.05) in at least one sample comparison were considered for biological networks. The networks were constructed by including interactions between differentially expressed mRNA and non-coding RNA and the connection of mRNA to biological functions of interest, including inflammation, immune response, fibrosis, melatonin signaling, senescence, and cell death. Interactions between mRNA were not included and the biological functions apoptosis, ferroptosis, and pyroptosis were aggregated into the cell death category to simplify network visualizations. Additionally, for biological functions with many genes, namely inflammation, a |log2FC|> 1 threshold was implemented in addition to the adjusted p-value threshold to further prioritize the visualized genes and keep the networks to a manageable size. The mRNA assignments to a given biological function were based on their presence within aggregated gene sets downloaded from MSigDB^79^ (Supplementary Table S3). All interaction networks were visualized using the open-source software Cytoscape (v3.10.2)^80^ *.*

### Data visualization

For mRNA, miRNA, and lncRNA analyses the pheatmap function from the R package ggplot2 was used to create heatmaps for the differentially expressed genes identified for each pairwise comparison (R software v3.3). The input values for each gene included in the heatmaps were scaled across samples and are presented as z-scores. A value of 0.0001 was added to the counts for any sample where counts were equal to 0 for a given gene to avoid undefined values upon log_2_ transformation. For heatmaps that highlight genes tied to specific biological functions, those genes were assigned to a given biological function based on the same aggregated gene sets downloaded from MSigDB as used in the constructed biological networks (Supplementary Table S3).

### Real time RT-PCR analysis

Individual qRT-PCR reactions were performed using QuantStudio 3 with RT2 First Strand Synthesis kit and RT2 SYBR Green qPCR Master Mix (QIAGEN). The following RNA primers were purchased from Qiagen, gene globe IDs are included for mRNA and assay IDs for non-coding RNA : CDKN1A (PPH00211E), GPX4 (PPH05586B), HSPB1 (PPH00165F), PTGS2 (PPH00165F), ACSL4 (PPH00165F), TFRC (PPH00990H), SQLE (PPH06371A), FANCD2 (PPH14413A), TGFB1 (PPH01904D), TIMP1 (PPH00771C), TLR4 (PPH01795F), CCN2 (PPH00550G), DINO^81^(FP-GCAATGGTGTGCCTGACTAT; RP-TTCTGGCTTCCCAGAG), GDF15(PPH01935C), PCNA (PPH00216B), MDM2 (PPH00193E), PHLDA3 (PPH15380B), BAX (PPH00078B), FOXM1 (PPH00943F), HIST1H3G (PPH14963A), LMNB1 (PPH00278B) and RPLP0 (PPH21138F), hsa-miR-122-5p (YD00619864), hsa-miR-21-5p (YD00619870), hsa-miR-34a-5p (YD00618250), hsa-miR-432-5p (YP00204776) and UniSp6 (YP00203954). QRT-PCR analysis was performed on select mRNA, lncRNA and miRNA. Relative expression was calculated as: 2^−dCt^ where dCt = Ct [test gene] − Ct [RPLP0 for mRNA and lncRNA or UniSp6 for miRNA]. Data analyzed with QuantStudio Design & Analysis software (v1.5.2).

### Cytotoxicity assay

CCK8 assay (Ab228554, abcam) was used to measure LSECs cytotoxicity after irradiation and NACA treatment in two-dimensional monolayer culture. LSECs were cultured in 96-well plates at a density of approximately 1.0 × 10^4^ cells per well for 24 h. The cells were then irradiated with 8 Gy and incubated with NACA (5.9–750 μM) for 24 h. CCK8 reagent was added to the cells and incubated at 37 °C for 2.5 h, followed by measuring the absorbance at 460 nm using a plate reader (Agilent, BioTek Synergy H1 Multimode Reader).

LDH-Glo™ Cytotoxicity Assay (Promega, J2380) was used to measure cell death in hepatocytes and NPCs in the quad culture liver chip. We followed the previously described protocol^70^. Briefly, effluents from the epithelial and endothelial channels were collected separately 3 days after radiation exposure and NACA treatment. To stabilize LDH activity, effluents were diluted 20-fold using LDH storage buffer, containing 20 mM Tris·HCl (pH 7.5), 10% (v/v) glycerol, and 1% (w/v) bovine serum albumin (BSA) and stored at 4 °C. The effluents were diluted twofold using LDH detection reagent and incubate at RT for 1 h, followed by measuring luminescence using the same plate reader.

### Immunofluorescence staining and microscopy

The fixation of cells in quad culture liver chip followed that previously described^27^. Briefly, cells in both channels were fixed with 1% paraformaldehyde (PFA) in warm medium and incubated at room temperature (RT) for 5 min. Subsequently, they were fixed with 4% PFA in PBS(+ Mg/ + Ca) for an additional 30 min at RT. This was Followed by washing with PBS (− Mg/ − Ca) and storage at 4 °C. For γ-H2AX staining, the cells were permeabilized with 1% Triton-X-100 in PBS for 30 min at 37 °C and 10 min at RT. For α-SMA staining, the cells were permeabilized with 1% Saponin in PBS for 30 min at RT. Followed by blocking with 1% BSA and 10% goat serum in PBS overnight at 4 °C on a shaker. The cells were then incubated with primary antibodies directed against γ-H2AX (Sigma, 05-636, dilution 1:100), CD31 (Abcam, ab215912, dilution 1:50), or α-SMA (Abcam, ab5694, dilution 1:1000) overnight at 4 °C on a shaker. Secondary antibodies (Abcam) were then introduced in the channels at RT for 2 h, followed by staining with DAPI (AB228549, DAPI) and Phalloidin (Abcam, AB176759). Microscopy was performed with confocal microscopy (Zeiss Axio Imager M2 with an Axiocam 712 mono camera) using Zen software (v3.4, Blue Edition) or automated cell imagers (Agilent, BioTek Lionheart FX automated microscope) using Gen5 software (v3.12). Quantification of the foci and the immunofluorescence images was performed using ImageJ Fiji software (v1.54 k).

### Biochemical assays

The Triglyceride Assay Kit (Abcam, Ab65336) was used to quantify the triglyceride levels in hepatocytes. Briefly, hepatocytes cell lysates were collected from the top channel 3 days after radiation exposure and NACA treatment using 5% NP-40/ddH_2_O solution and stored at − 80 °C. To prevent cross-contamination, cell lysis for protein extraction was performed as in the RNA isolation method. Triglyceride Assay was performed as outlined by vendor-provided protocols. After thawing, the cell lysates were heated and cooled down to RT to solubilize all triglycerides. The cell lysates were then diluted 1:10 in ddH_2_O and mixed with Triglyceride Reaction mix. Fluorescence at Ex/Em = 535/587 nm was measured using the Agilent BioTek reader and Gen5 software (v3.11).

Human Urea assay kit (Sigma–Aldrich, MAK006) and Albumin ELISA Kit (Abcam, ab179887) were used to quantify hepatocytes albumin and urea levels followed that previously described^26^. Briefly, effluent from the top channel was collected 3 days after radiation exposure and NACA treatment and stored at − 80 °C. For the Urea assay, effluents were thawed and diluted 1:5 in assay buffer and mixed with the kit’s Reaction Mix. Absorbance at 570 nm was measured using the Agilent BioTek plate reader. For the Albumin assay, effluents were thawed and diluted 1:5 in assay buffer and mixed with Antibody Cocktail. Absorbance at 450 nm was measured using the Agilent BioTek plate reader.

The V-PLEX Vascular Injury Panel 2 Human Kit (Meso Scale Diagnostics, K15198G) was used to quantify the level of VCAM-1, ICAM-1, SAA and CRP in NPCs. Human IL-6 ELISA Kit (Abcam, ab46042), Human Thrombomodulin ELISA Kit (Abcam, ab214029), and Human PDGF BB ELISA Kit (Abcam, ab100624) were used to quantify the levels of IL-6, Thrombomodulin and PDGFBB in NPCs. Briefly, NPCs cell lysates were collected from the bottom channel 3 days after radiation exposure and NACA treatment using Extraction Buffer 5× PTR (Abcam, ab193970) and stored at − 80 °C. For V-PLEX Vascular Injury Panel 2 Human Kit, after thawing, effluents were added to plates at a 1:2 dilution in assay buffer and mixed with Antibody solution. Plates were read on the MESO QuickPlex SQ 120 (Meso Scale Discoveries) using MSD Discovery Workbench software (v 4.0). For IL-6, Thrombomodulin and PDGFBB ELISA Kits, after thawing, effluents were added to plates at a 1:10 dilution for IL-6, 1:4 dilution for Thrombomodulin or 1:4 dilution for PDGFBB in assay buffer and mixed with Antibody solution. Absorbance at 450 nm was measured using the Agilent BioTek plate reader using Gen5 software (v3.11).

### Statistical analysis

All experiments were carried out at n = 3–6 (see figure captions), and results and error bars in this article are presented as mean ± standard error of the mean. Data analysis and graph plotting were performed on GraphPad Prism software (v10). For multiple comparisons, one way ANOVA was used with Tukey or Dunnet’s post-hoc tests. All n, p-values, F values, and df values are mentioned in the respective figure or the associated legends.

## Supplementary Information

Supplementary Information 1.

Supplementary Information 2.

Supplementary Information 3.

Supplementary Information 4.

Supplementary Information 5.

## Data Availability

All data analyzed during this study has been submitted to the NLM/NCBI SRA database accession PRJNA1171420.
