# Supplementary material for: Liver quad culture chip as a model for radiation injury research
Source: Sci Rep. 2025 Apr 11;15:12414. doi: 10.1038/s41598-025-96140-1 (PMC11992238; doi:10.1038/s41598-025-96140-1)
Supplement: Supplementary file 3 — Supplementary Information 3. [file 41598_2025_96140_MOESM3_ESM.docx]

**Supplemental Figure legends Liver quad-culture**

**Supplemental Table S1: Statistically significant genes in hepatocytes**. Statistically significant mRNA and lncRNA across all doses and times are shown for hepatocytes. Data includes common name (geneid), adjusted pvalue (padj), log2FC and comparison group.

**Supplemental Table S2: Statistically significant genes in NPCs**. Statistically significant mRNA and lncRNA across all doses and times are shown for NPCs. Data includes common name (geneid), adjusted pvalue (padj), log2FC and comparison group.

**Supplemental Figure S1 RT-PCR validation of key radiation response genes in liver quad-culture chip after radiation injury**. PCR analysis was performed for genes previously discovered in our liver co-culture model in hepatocytes and NPCs 6h post radiation injury. Radiation doses used include 0 Gy, 4 Gy, 6 Gy, 8 Gy and 10 Gy. All hepatocyte samples were performed with n=4 except 8 Gy is n=3. All NPCs samples were performed with n=4 except 8 Gy and 10 Gy is n=3.

HEP CDKN1A, GDF15, MDM2, PHLDA3, BAX mRNA level. n=3, one-way ANOVA with Dunnett’s test, significance at p<0.05, F(CDKN1A)=55.31, F(GDF15)=20.10,F(MDM2)=36.80, F(PHLDA3)=4.588, F(BAX)=6.650, DF=4. NPC CDKN1A, GDF15, MDM2, FOXM1, HIST1H3G mRNA level. n=3, one-way ANOVA with Dunnett’s test, significance at p<0.05, F(CDKN1A)=29.95, F(GDF15)=5.131, F(MDM2)=17.88, F(FOXM1)=7.205, F(HIST1H3G)=5.381, DF=4.

**Supplemental Figure S2 Volcano plots for each dose and time for mRNA and lncRNA**. Volcano plots for 6h, 24h and 7 day after 4 Gy or 8 Gy radiation in hepatocytes or NPCs. Black indicates no significance. Blue indicates downregulation and red indicates upregulation with statistical significance (abs)log2FC > 1, padj < 0.05).

**Supplemental Figure S3 PCA plots demonstrate dose specific changes in mRNA.** PCA plots for hepatocyte and NPCs mRNA and lncRNA for all doses and times are shown to understand how different treatments impacted samples overall. We observed a time specific clustering in NPCs mRNA and a dose specific clustering in hepatocyte mRNA.

**Supplemental Figure S4 Histone expression is altered by radiation injury**. Individual gene plots for sample histones for Hepatocytes (S3A) and NPCs (S3B) show changes in histone expression that tend to remain at the 7d time point. Further histone expression changes are noted in Supplemental Table S3 for NPCs. Only 3 histones showed significant dysregulation for hepatocytes and these are shown.

**Supplemental Figure S5 Statistically significant miRNA as determined by Novogene.** Statistically significant miRNA are shown (padj < 0.05) for all doses and times. miRNA name, log2FC and padj. Sequencing data is shown for miR-34a-5p in hepatocytes at 7 days post injury and miR-432-5p in NPCs at 7 days post injury.

**Supplemental Figure S6 Heatmap for Fibrosis related genes across dose and time indicate potential fibrosis markers**. Supplemental Figure S6A shows the hepatocyte heatmap for fibrosis while Supplemental Figure S6B shows the NPCs heatmap for fibrosis, for each dose and time point. Red indicates upregulation and blue indicates downregulation with color scheme based on log2FC. Arrows indicate genes which show upregulation in both hepatocytes and NPCs in 8 Gy samples compared to 0 Gy samples at 7d. The highest bars represent time: Green represents 6h, light blue represents 24h and yellow represent 7d. The second bars represent dose where gray represents 0Gy, pink indicates 4 Gy and orange indicates 8 Gy.

**Supplemental Figure S7 Liver quad-culture NPCs show more gene expression changes relevant to fibrosis, senescence and cytokine storm compared to Liver co-culture LSECs**. We compared gene expression in LSECs in co-culture chip and NPCs in quad -culture chip, the “support cells” of the liver, after radiation injury using IPA. The only dose shared between the two experimental models was 4 Gy. Canonical analysis (S7A) of 4Gy 24h LSECs and 4 Gy 24h NPCs is shown with orange indicating anticipated pathway activation and blue indicating anticipated pathway inhibition. We then looked at gene heatmaps within IPA to understand which genes showed changes for either experimental model. We focused on senescence (S7B), Hepatic fibrosis signaling (S7C), and Pathogen induced cytokine storm signaling (S7C). Where red indicates upregulation and green indicates downregulation.

**Supplemental Figure S8 DNA repair pathways in hepatocytes shows multiple genes are upregulated after ionizing radiation.** Genes relevant to homologous recombination, non-homologous end-joining, base excision repair, nucleotide excision repair, pyrimidine biosynthesis and purine biosynthesis are shown. Log2fold change is shown on the X axis why -log(pvalue) is shown on a color gradient with red indicating a lower p-value.

**Supplemental Figure S9 DNA repair pathways in NPCs show downregulation of multiple genes after ionizing radiation**. Genes relevant to homologous recombination, non-homologous end-joining, base excision repair, nucleotide excision repair, pyrimidine biosynthesis and purine biosynthesis are shown. Log2fold change is shown on the X axis why -log(pvalue) is shown on a color gradient with red indicating a lower p-value.

**Supplemental Figure S10 NACA modifies radiation induced gene changes that impact DNA damage and stress response pathway in hepatocytes and NPCs.** RT-PCR results show that NACA alters expression of DNA damage and stress response markers: DINO, PCNA, MDM2 and LMNB1 compared to radiation alone in hepatocytes and NPCs. HEP DINO, PCNA, MDM2 and LMNB1 mRNA level. 6h after radiation, n=3, one-way ANOVA with Tukey’s test, significance at p<0.05, F(DINO)=12.49, F(PCNA)=50.24 , F(MDM2)=69.24 , F(LMNB1)=6.616 , DF=2. NPC DINO, PCNA, MDM2 and LMNB1 mRNA level. 6h after radiation, n=3, one-way ANOVA with Tukey’s test, significance at p<0.05, F(DINO)=7.507, F(PCNA)=50.24, F(MDM2)=29.71, F(LMNB1)=185.3, DF=2.

**Supplemental Figure S11 NACA modifies radiation induced gene changes that impact Ferroptosis pathway in NPCs.** RT-PCR results show the impact of NACA on ferroptosis markers at 3 days post radiation exposure in NPCs with white indicating 0 Gy, gray indicating 8Gy+NACA and black indicating 8 Gy. Figure 7C shows the impact of NACA on ferroptosis markers at 3 days post radiation exposure in NPCs with white indicating 0 Gy, gray indicating 8Gy+NACA and black indicating 8 Gy. Ferroptosis related genes (GPX4, HSPB1) level was calculated 6h after radiation, n=3, one-way ANOVA with Tukey’s test, significance at p<0.05, F(GPX4)=11.43, F(HSPB1)=15.79, DF=2. Ferroptosis related genes (PTGS2, ACSL4, TFRC, SQLE, FANCD2) levels were calculated 3 days after radiation, n=3, one-way ANOVA with Tukey’s test, significance at p<0.05, F(PTGS2)=15.75, F(ACSL4)=12.64, F(TFRC)=5.599, F(SQLE)=24.65, F(FANCD2)=13.56, F(miR-432-5p)=6.235, DF=2.
