# Supplementary material for: Liver quad culture chip as a model for radiation injury research
Source: Sci Rep. 2025 Apr 11;15:12414. doi: 10.1038/s41598-025-96140-1 (PMC11992238; doi:10.1038/s41598-025-96140-1)
Supplement: Supplementary file 4 — Supplementary Information 4. [file 41598_2025_96140_MOESM4_ESM.pptx]

## Slide 1
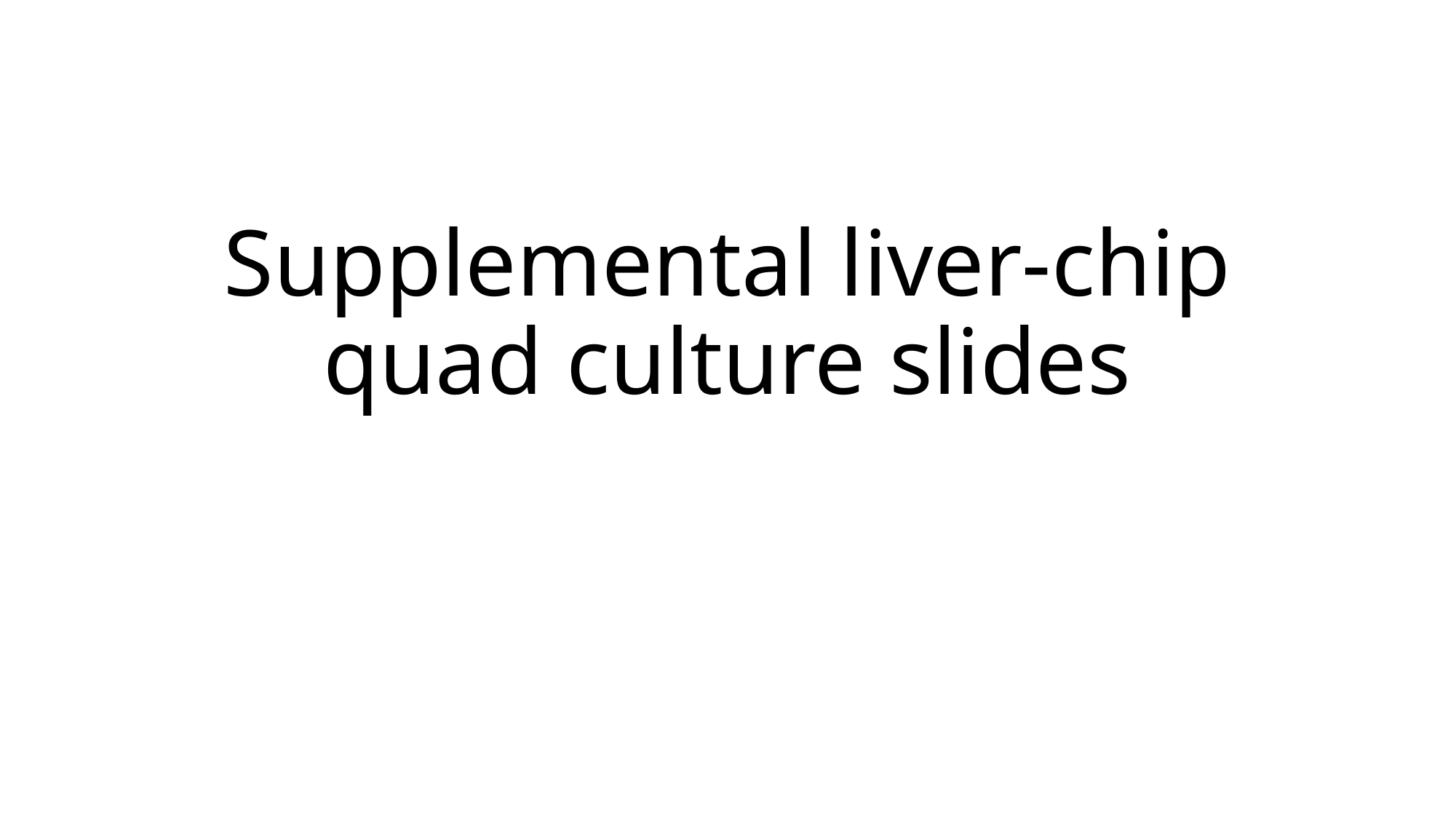

# Supplemental liver-chip quad culture slides

## Slide 2
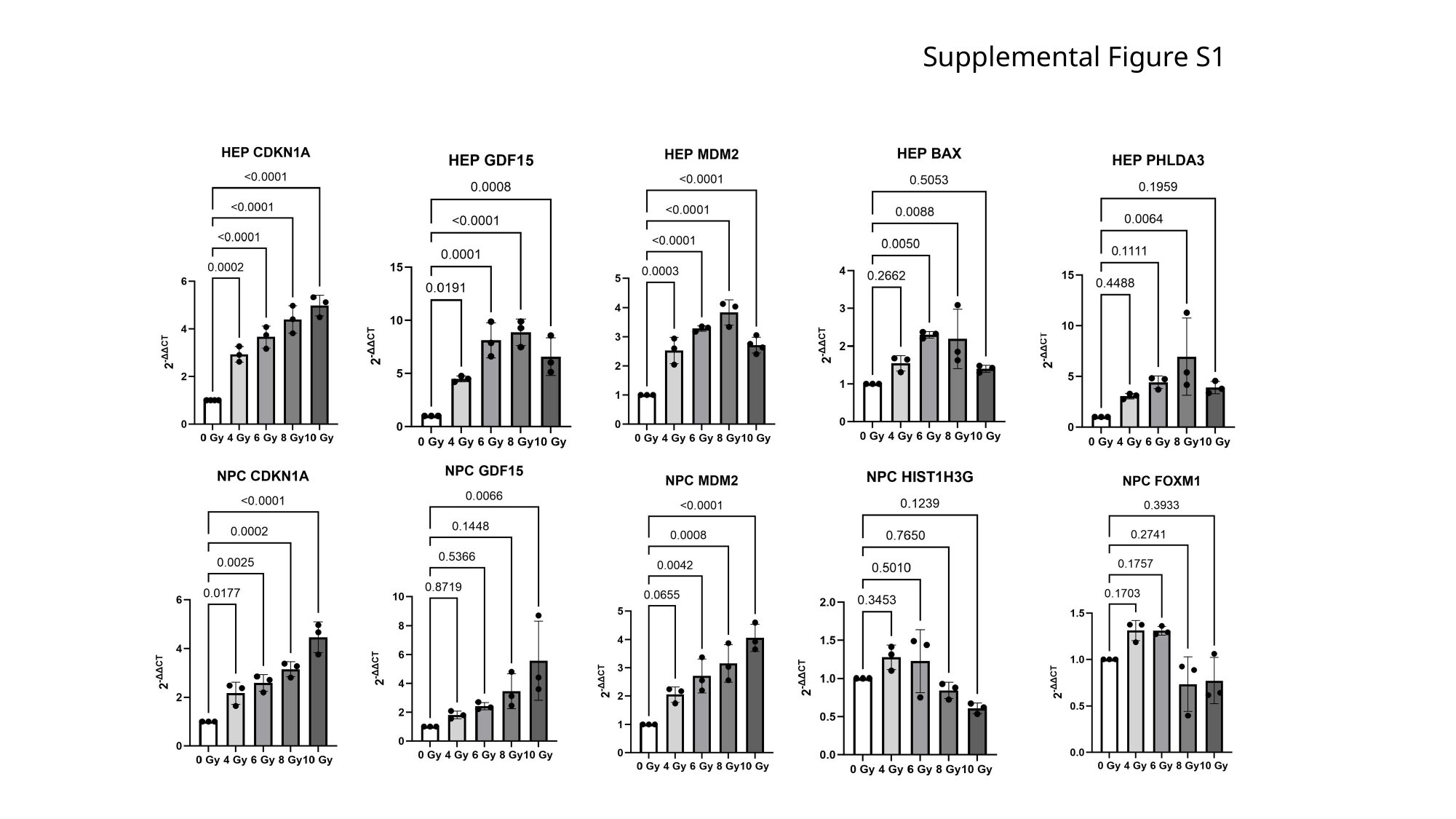

Supplemental Figure S1

## Slide 3
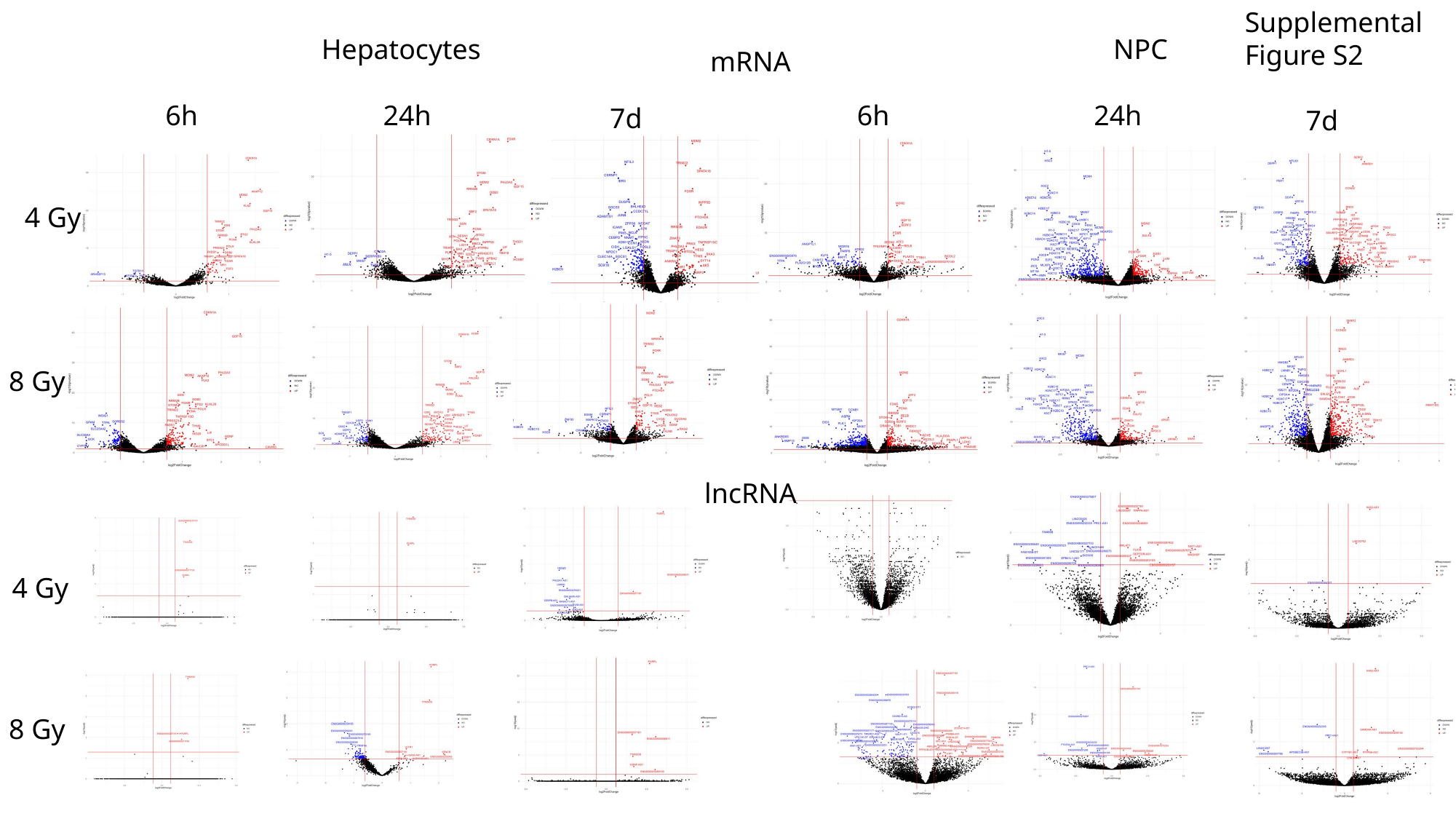

Supplemental
Figure S2
Hepatocytes
NPC
mRNA
6h
24h
6h
24h
7d
7d
4 Gy
8 Gy
lncRNA
4 Gy
8 Gy

## Slide 4
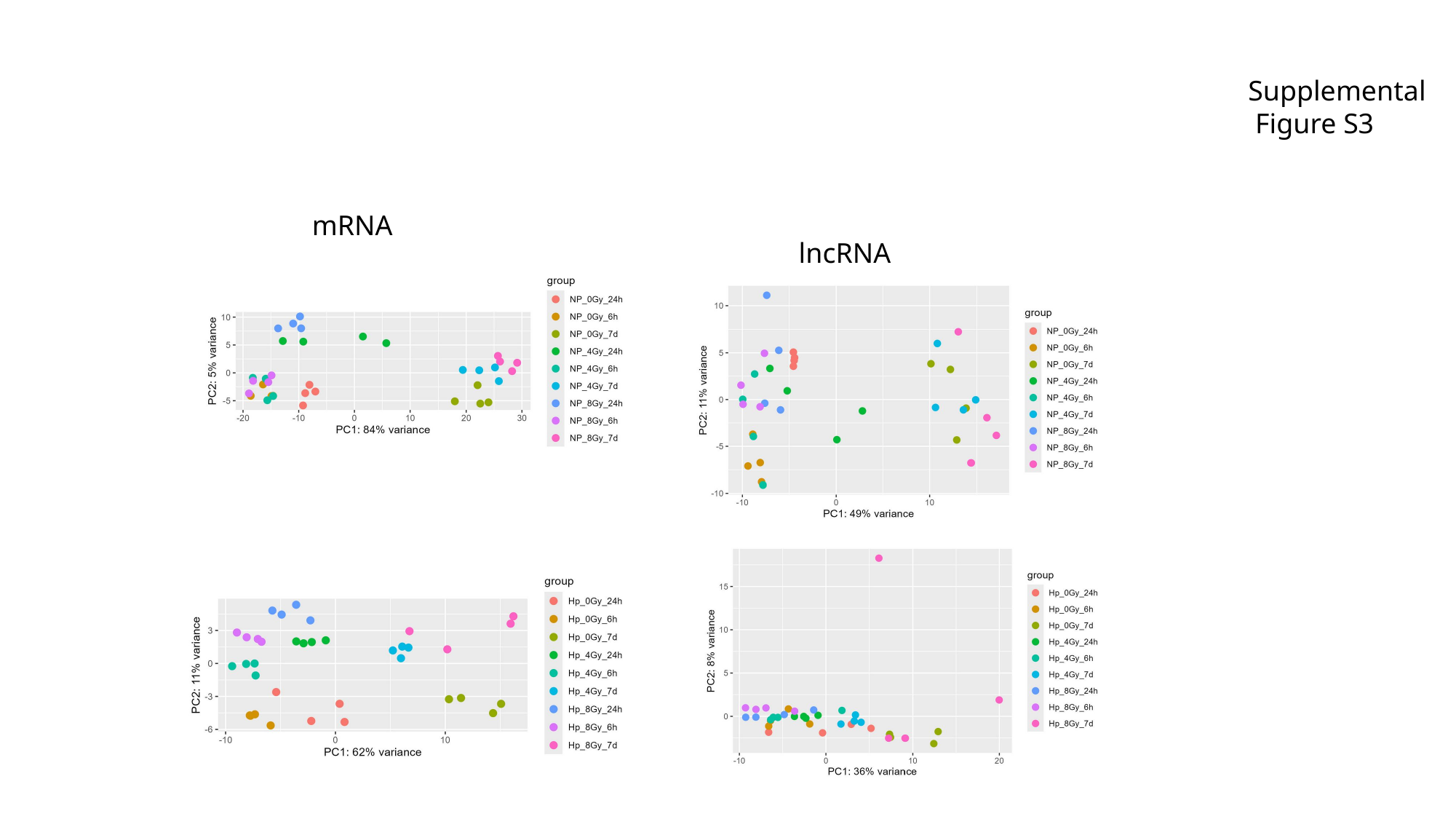

Supplemental
 Figure S3
mRNA
lncRNA

## Slide 5
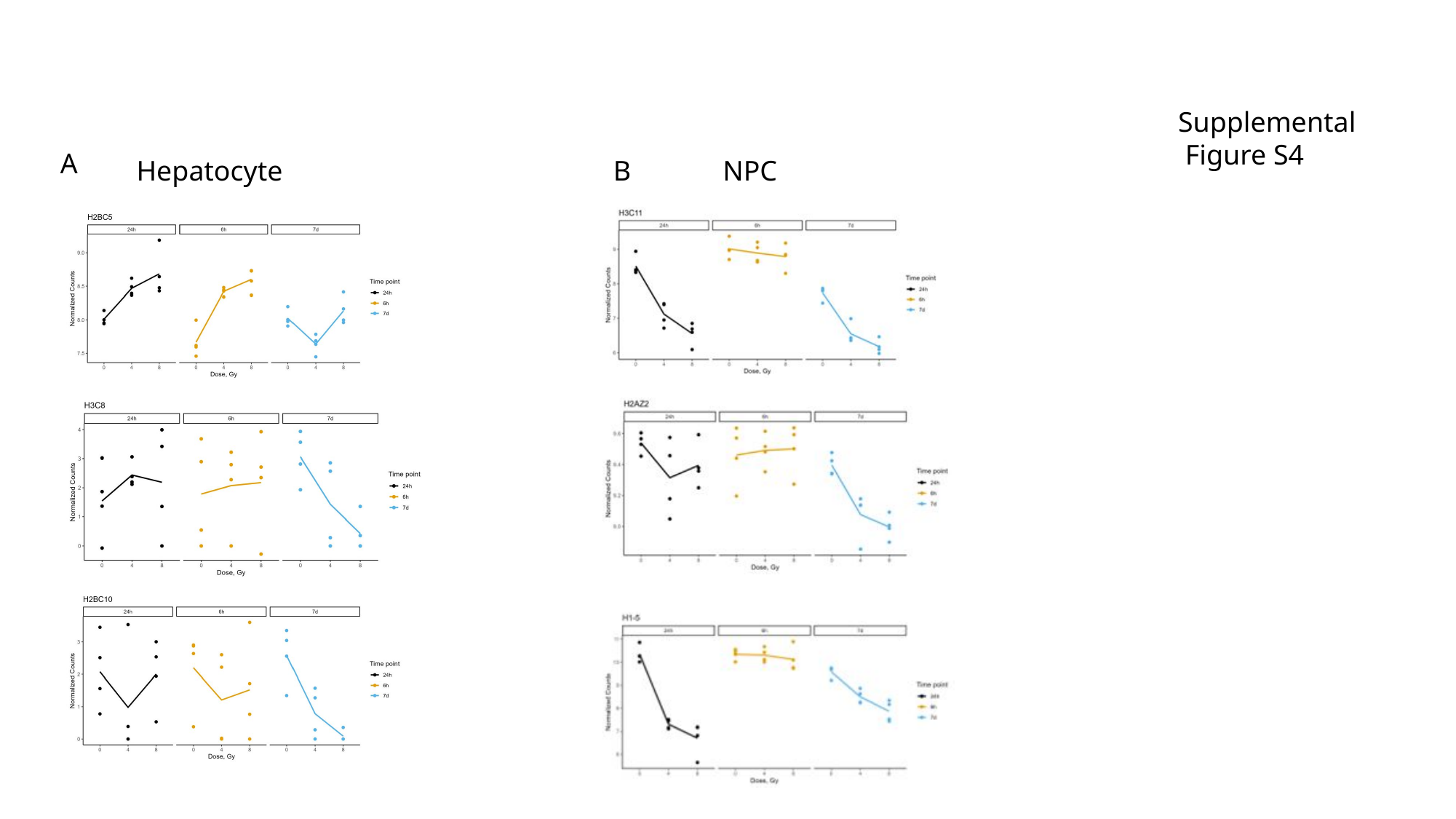

Supplemental
 Figure S4
A
Hepatocyte
B
NPC

## Slide 6
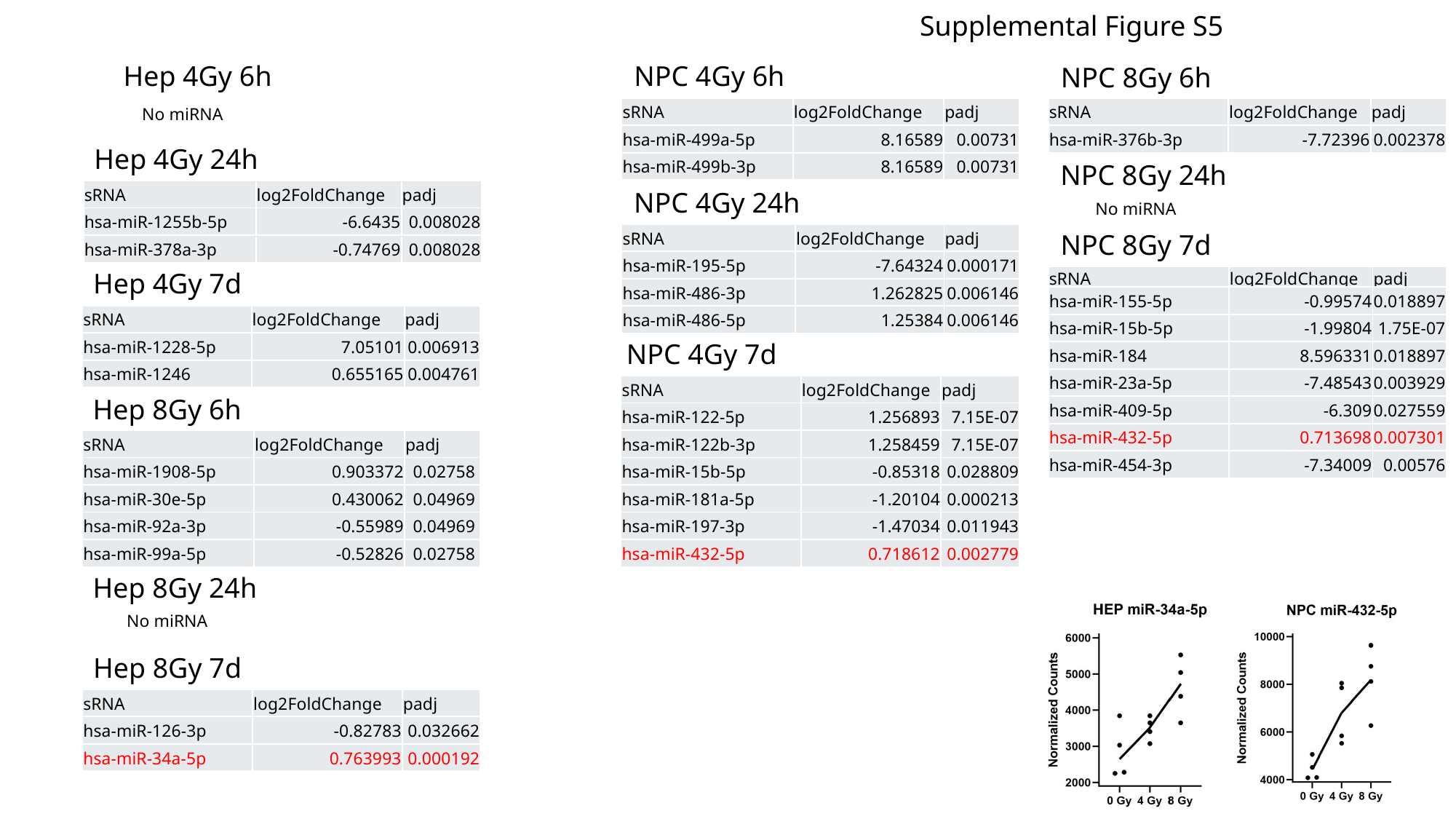

Supplemental Figure S5
Hep 4Gy 6h
NPC 4Gy 6h
NPC 8Gy 6h
| sRNA | log2FoldChange | padj |
| --- | --- | --- |
| hsa-miR-499a-5p | 8.16589 | 0.00731 |
| hsa-miR-499b-3p | 8.16589 | 0.00731 |
| sRNA | log2FoldChange | padj |
| --- | --- | --- |
| hsa-miR-376b-3p | -7.72396 | 0.002378 |
No miRNA
Hep 4Gy 24h
NPC 8Gy 24h
NPC 4Gy 24h
| sRNA | log2FoldChange | padj |
| --- | --- | --- |
| hsa-miR-1255b-5p | -6.6435 | 0.008028 |
| hsa-miR-378a-3p | -0.74769 | 0.008028 |
No miRNA
NPC 8Gy 7d
| sRNA | log2FoldChange | padj |
| --- | --- | --- |
| hsa-miR-195-5p | -7.64324 | 0.000171 |
| hsa-miR-486-3p | 1.262825 | 0.006146 |
| hsa-miR-486-5p | 1.25384 | 0.006146 |
Hep 4Gy 7d
| sRNA | log2FoldChange | padj |
| --- | --- | --- |
| hsa-miR-155-5p | -0.99574 | 0.018897 |
| hsa-miR-15b-5p | -1.99804 | 1.75E-07 |
| hsa-miR-184 | 8.596331 | 0.018897 |
| hsa-miR-23a-5p | -7.48543 | 0.003929 |
| hsa-miR-409-5p | -6.309 | 0.027559 |
| hsa-miR-432-5p | 0.713698 | 0.007301 |
| hsa-miR-454-3p | -7.34009 | 0.00576 |
| sRNA | log2FoldChange | padj |
| --- | --- | --- |
| hsa-miR-1228-5p | 7.05101 | 0.006913 |
| hsa-miR-1246 | 0.655165 | 0.004761 |
NPC 4Gy 7d
| sRNA | log2FoldChange | padj |
| --- | --- | --- |
| hsa-miR-122-5p | 1.256893 | 7.15E-07 |
| hsa-miR-122b-3p | 1.258459 | 7.15E-07 |
| hsa-miR-15b-5p | -0.85318 | 0.028809 |
| hsa-miR-181a-5p | -1.20104 | 0.000213 |
| hsa-miR-197-3p | -1.47034 | 0.011943 |
| hsa-miR-432-5p | 0.718612 | 0.002779 |
Hep 8Gy 6h
| sRNA | log2FoldChange | padj |
| --- | --- | --- |
| hsa-miR-1908-5p | 0.903372 | 0.02758 |
| hsa-miR-30e-5p | 0.430062 | 0.04969 |
| hsa-miR-92a-3p | -0.55989 | 0.04969 |
| hsa-miR-99a-5p | -0.52826 | 0.02758 |
Hep 8Gy 24h
No miRNA
Hep 8Gy 7d
| sRNA | log2FoldChange | padj |
| --- | --- | --- |
| hsa-miR-126-3p | -0.82783 | 0.032662 |
| hsa-miR-34a-5p | 0.763993 | 0.000192 |

## Slide 7
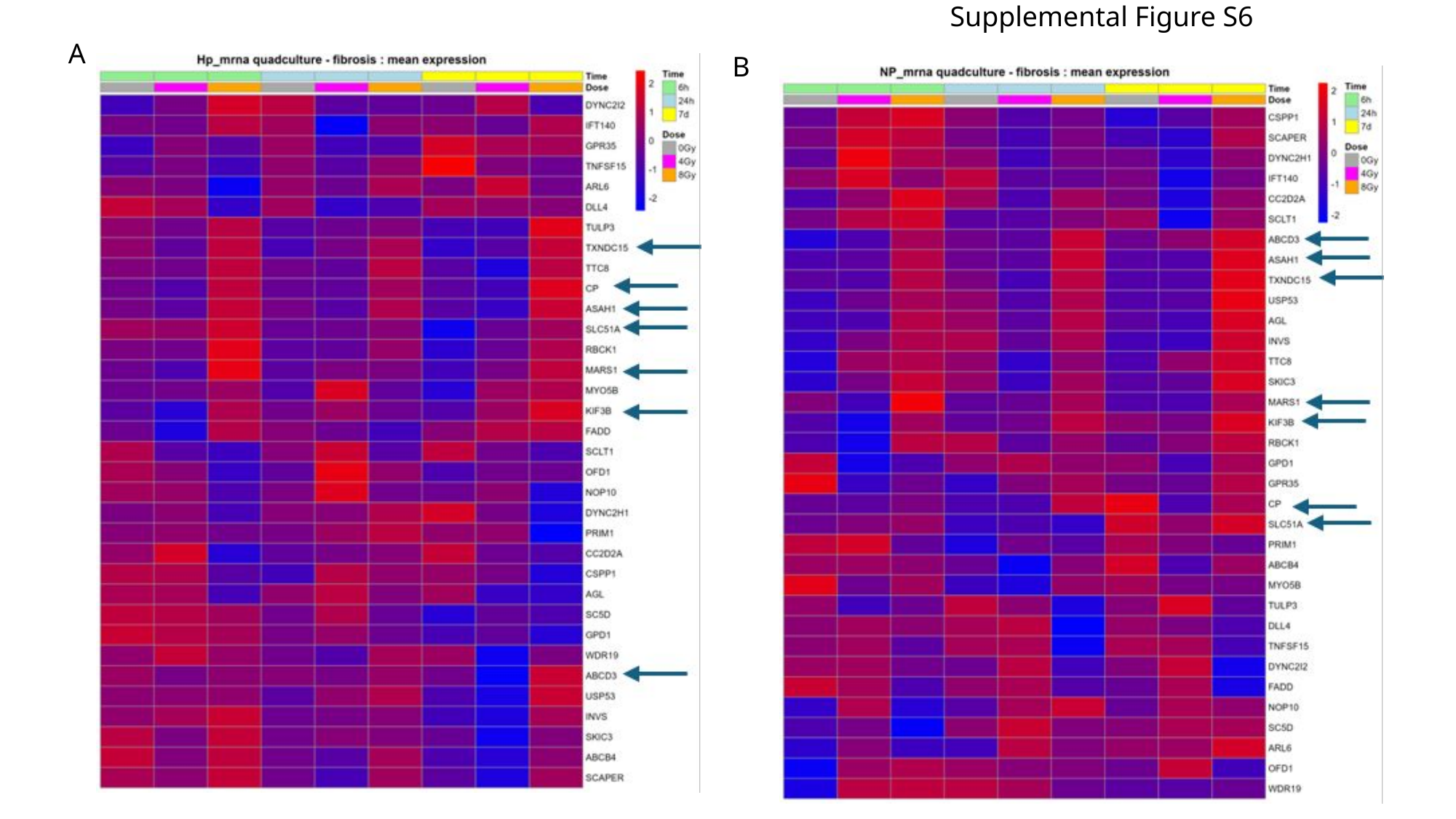

Supplemental Figure S6
A
B

## Slide 8
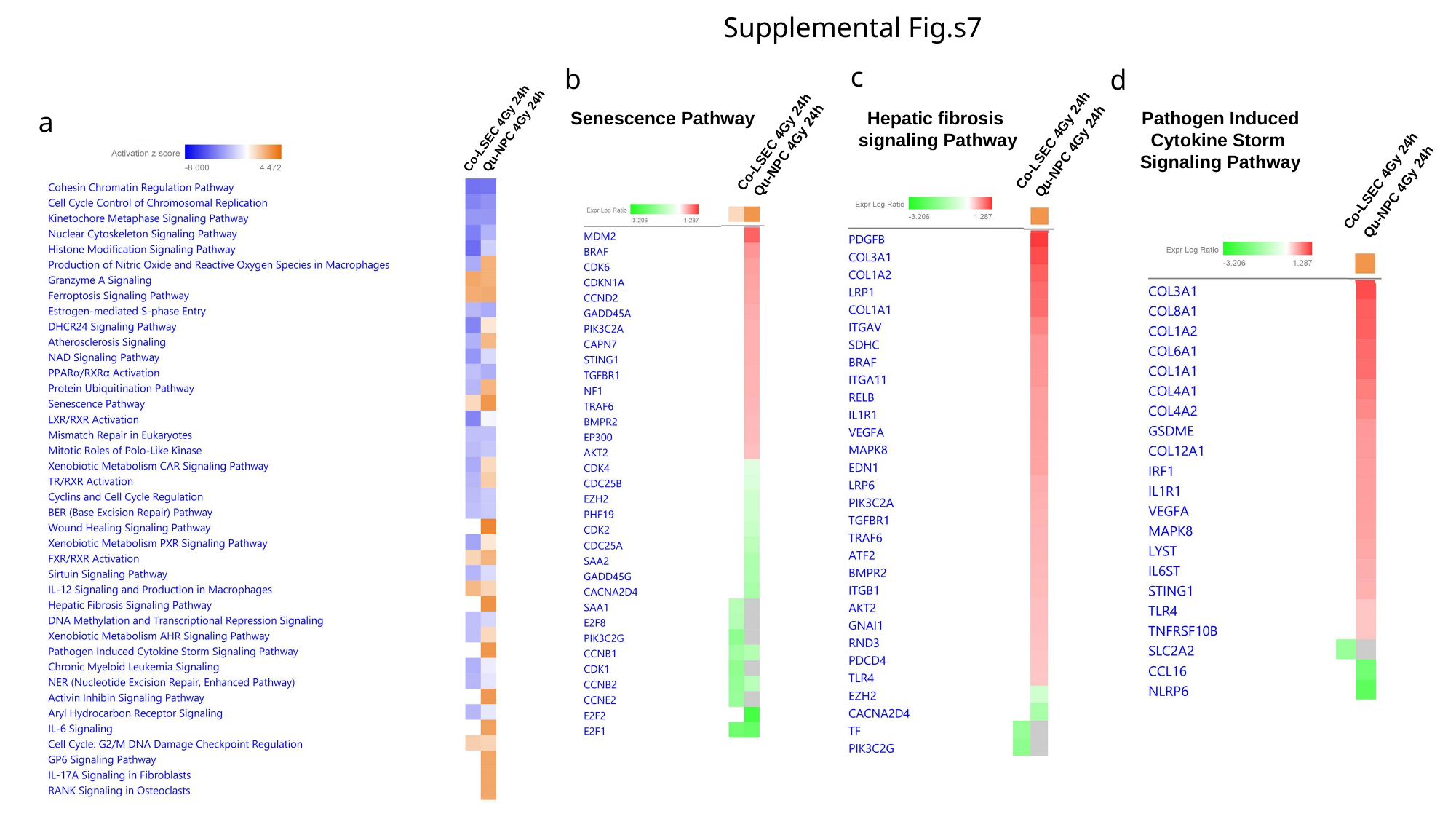

Supplemental Fig.s7
c
b
d
Co-LSEC 4Gy 24h
Qu-NPC 4Gy 24h
Co-LSEC 4Gy 24h
Qu-NPC 4Gy 24h
Co-LSEC 4Gy 24h
Qu-NPC 4Gy 24h
a
Senescence Pathway
Hepatic fibrosis
signaling Pathway
Pathogen Induced
Cytokine Storm
Signaling Pathway
Co-LSEC 4Gy 24h
Qu-NPC 4Gy 24h

## Slide 9
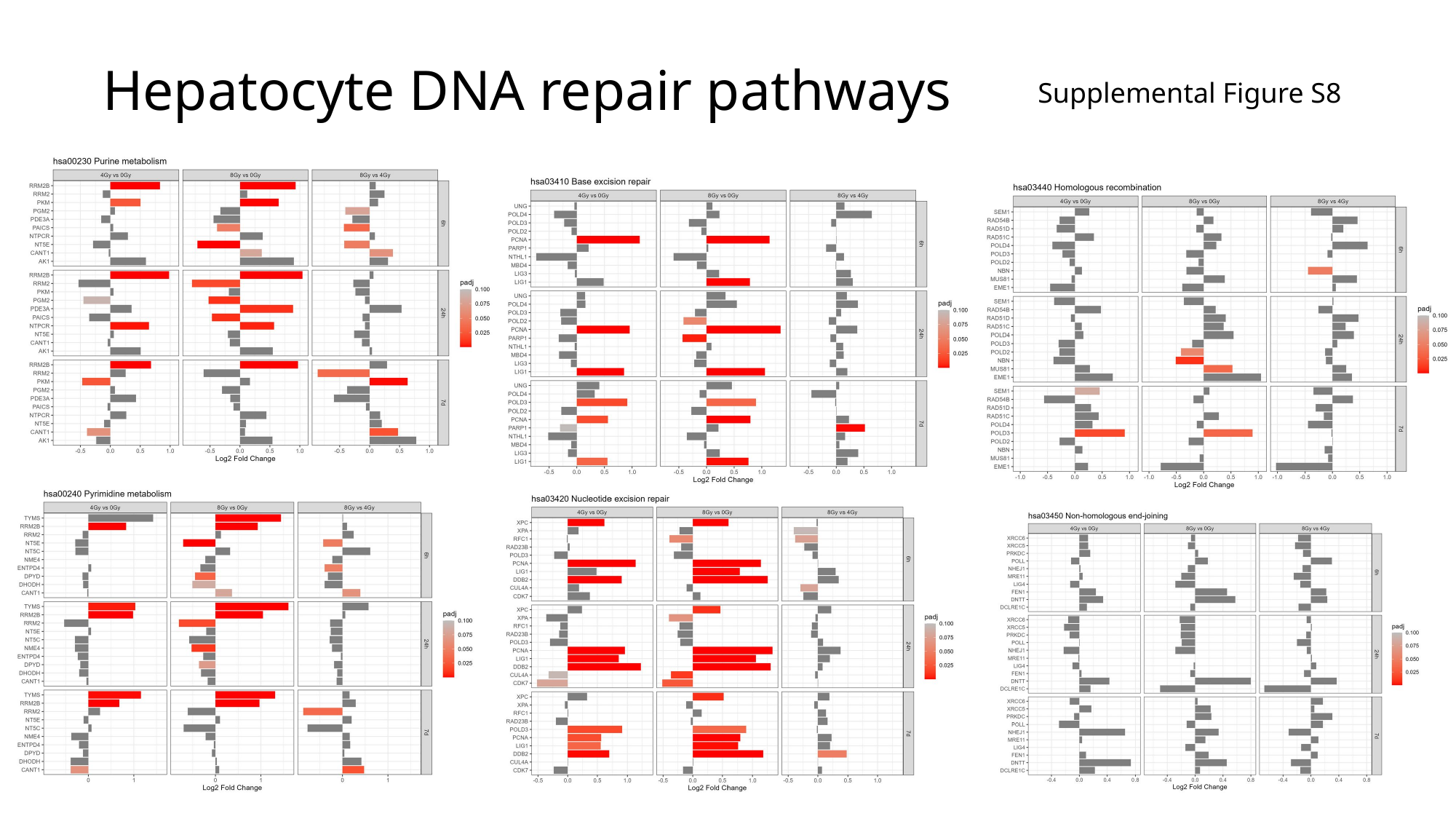

# Hepatocyte DNA repair pathways
Supplemental Figure S8

## Slide 10
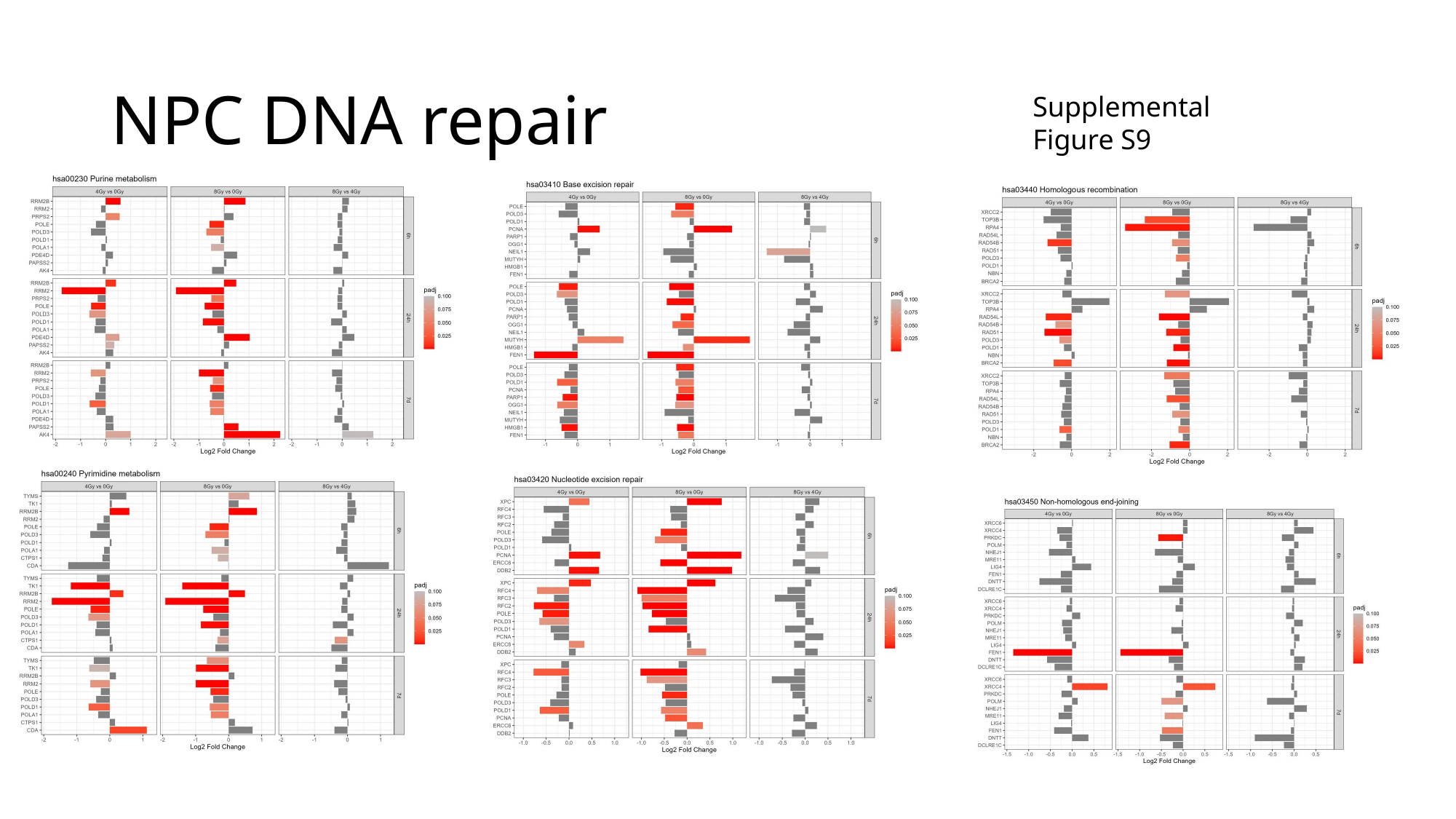

# NPC DNA repair
Supplemental
Figure S9

## Slide 11
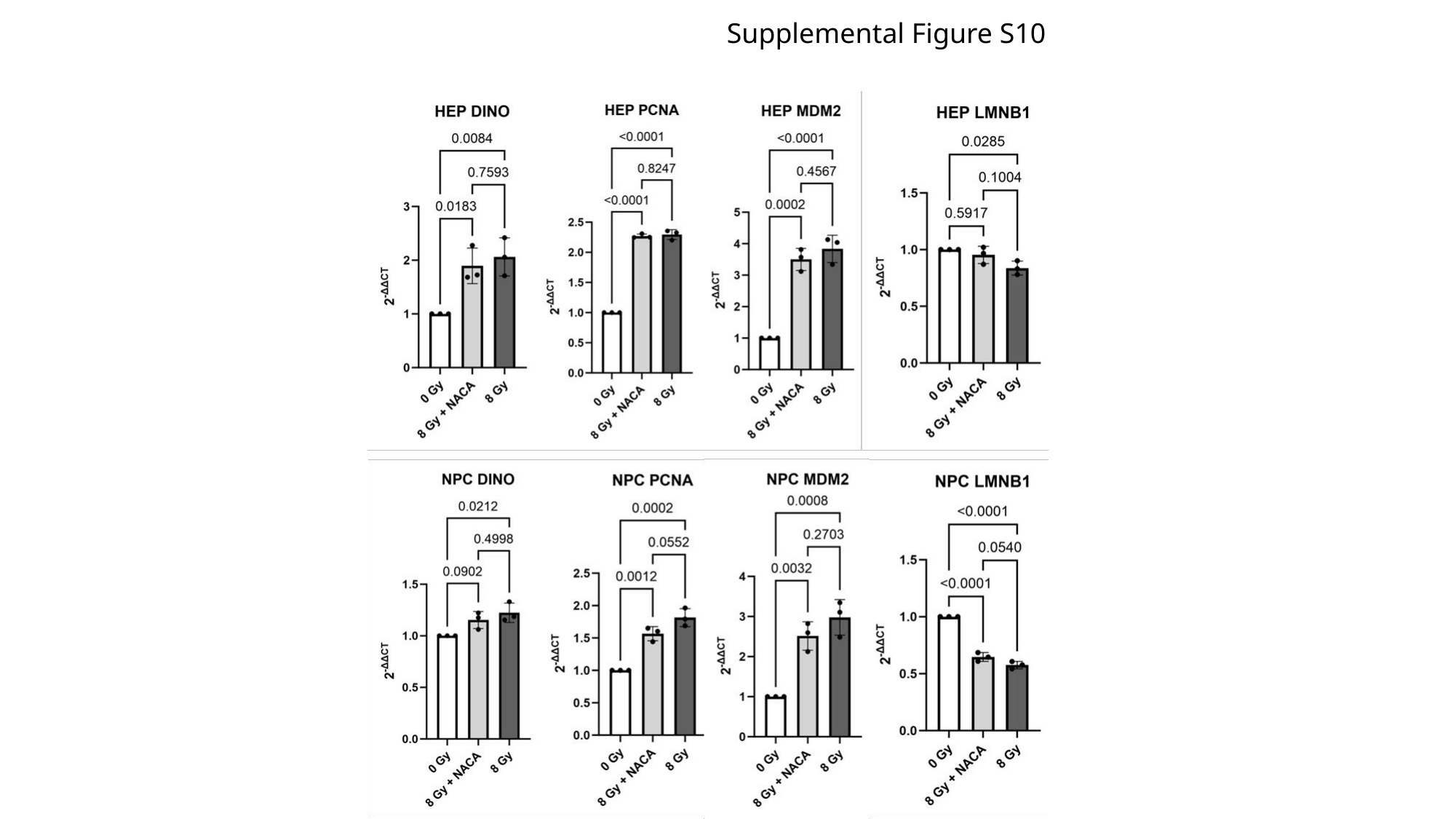

Supplemental Figure S10

## Slide 12
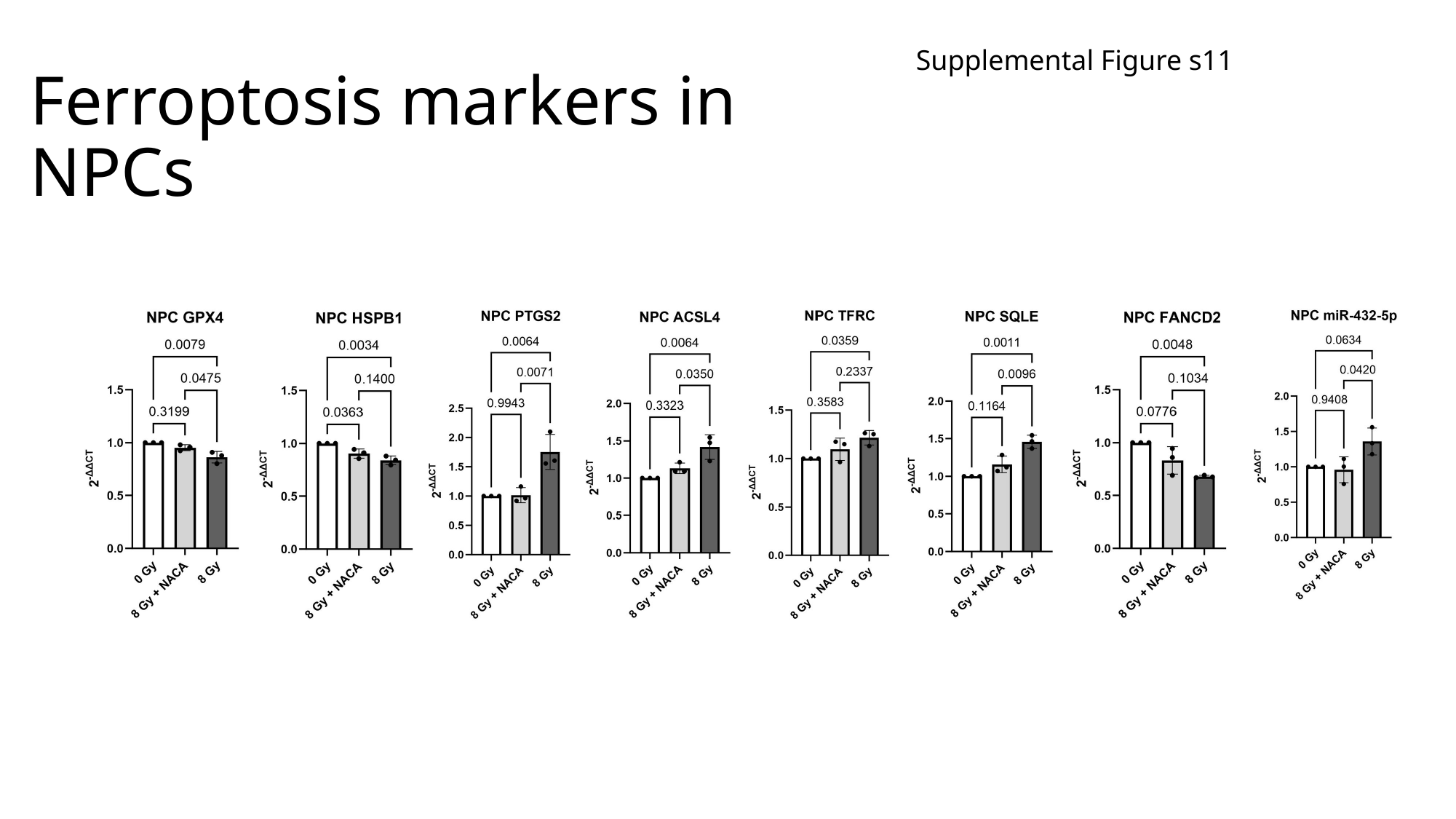

Supplemental Figure s11
# Ferroptosis markers in NPCs
